# Transcriptome analysis reveals mechanisms of geroprotective effects of fucoxanthin in *Drosophila*

**DOI:** 10.1186/s12864-018-4471-x

**Published:** 2018-02-09

**Authors:** Alexey Moskalev, Mikhail Shaposhnikov, Nadezhda Zemskaya, Alexey Belyi, Eugenia Dobrovolskaya, Anna Patova, Zulfiya Guvatova, Elena Lukyanova, Anastasiya Snezhkina, Anna Kudryavtseva

**Affiliations:** 1 Engelhardt Institute of Molecular Biology, Russian Academy of Sciences, Moscow, Russia; 2 Institute of Biology of Komi Science Center of Ural Branch of Russian Academy of Sciences, Syktyvkar, Russia

**Keywords:** Fucoxanthin, Lifespan, Gene expression, Stress resistance, Locomotor activity, Fertility, *Drosophila melanogaster*

## Abstract

**Background:**

We have previously showed that the carotenoid fucoxanthin can increase the lifespan in *Drosophila melanogaster* and *Caenorhabditis elegans*. However, the molecular mechanisms of the geroprotective effect of fucoxanthin have not been studied so far.

**Results:**

Here, we studied the effects of fucoxanthin on the *Drosophila* aging process at the molecular and the whole organism levels. At the organismal level, fucoxanthin increased the median lifespan and had a positive effect on fecundity, fertility, intestinal barrier function, and nighttime sleep. Transcriptome analysis revealed 57 differentially expressed genes involved in 17 KEGG (Kyoto Encyclopedia of Genes and Genomes) pathways. Among the most represented molecular pathways induced by fucoxanthin, a significant portion is related to longevity, including MAPK, mTOR, Wnt, Notch, and Hippo signaling pathways, autophagy, translation, glycolysis, oxidative phosphorylation, apoptosis, immune response, neurogenesis, sleep, and response to DNA damage.

**Conclusions:**

Life-extending effects of fucoxanthin are associated with differential expression of longevity-associated genes.

**Electronic supplementary material:**

The online version of this article (10.1186/s12864-018-4471-x) contains supplementary material, which is available to authorized users.

## Background

Fucoxanthin is a one of the most abundant marine carotenoids, which is widely distributed in brown algae of the genera *Alaria, Cladosiphon, Cystoseira, Eisenia, Fucus, Hijikia, Ishige, Kjellmaniella, Laminaria, Myagropsis, Padina, Petalonia, Sargassum, Turbinaria, Undaria* and others [1, 2], as well as in diatoms *Phaeodactylum tricornutum* and *Isochrysis galbana* [1, 3]*.* Fucoxanthin contributes more than 10% of the estimated total production of carotenoids in nature [4]. Fucoxanthin may be one of the components of the so called “functional foods” that supplies the body with the needed amount of vitamins, fats, proteins, carbohydrates, etc., required for its healthy survival and prevent life-style-related diseases, like metabolic syndrome [5, 6].

In previous study on *Drosophila melanogaster* and *Caenorhabditis elegans* we have established that fucoxanthin can increase the lifespan of both models [7]. Fucoxanthin also increased the resistance to oxidative stress, had a positive effect on locomotor activity and activates the expression of some stress-resistance genes in *Drosophila*.

This paper aims to elucidate the molecular mechanisms of the geroprotective activity of fucoxanthin by total RNA sequencing. Transcriptome analysis revealed that geroprotective effect of fucoxanthin associatiated with differential expression of genes involved in longevity regulating pathways, autophagy, translation, glycolysis, oxidative phosphorylation, apoptosis, immune response, neurogenesis, sleep, and response to DNA damage. Among the most represented molecular pathways induced by fucoxanthin treatment, a significant portion is related to longevity, including MAPK, mTOR, Wnt, Notch, and Hippo signaling pathways. At the level of the whole organism, along with an increase of median lifespan, we observed a positive effect of fucoxanthin on fecundity and fertility, intestinal barrier function, and nighttime sleep in old individuals. Thus, the lifespan and healthspan extending effects of fucoxanthin at the organismal level is associated with differential expression of genes and changes in the activity of molecular pathways.

## Methods

### Treatment with fucoxanthin

*Drosophila melanogaster* wild type *Canton-S* line was obtained from Bloomington Stock Center at Indiana University (#1, Bloomington, USA). Flies were maintained on a yeast medium with a spread of yeast paste (50 g of inactivated by heating dry yeast per 60 ml of water). Starting from the first day of life, imagoes were fed by a yeast paste with fucoxanthin (Merck, USA) in concentration of 1 μM. According to our previous studies, the 1 μM had the most pronounced geroprotective effect on female flies in comparison with 0.3 and 0.5 μM [7]. Control animals were fed by yeast paste without fucoxanthin. Flies were transferred to a fresh medium twice a week.

### Lifespan analysis

Control and experimental flies were collected during 24 h after imago hatching, sorted by sex under carbon dioxide (CO_2_) anesthesia (Genesee Scientific, USA), and maintained in a constant climate chamber Binder KBF720-ICH (Binder, Germany) at 25 °C and 60% humidity in a 12:12 h light-dark cycle. The flies were housed in *Drosophila* narrow vials (Genesee Scientific, USA) at a density of 30 individuals per vial, with 5 vials per experimental variant. Dead flies were recorded daily. Lifespan experiments were performed in 2 replicates. The median lifespan, the age of 90% mortality (maximum lifespan), and the mortality rate doubling time (MRDT) were calculated. To compare the statistical differences in survival functions and median lifespan between control and experimental groups, the modified Kolmogorov-Smirnov and Gehan-Breslow-Wilcoxon tests were used, respectively [8, 9]. A Wang-Allison test was used to estimate the differences in the age of 90% mortality [10]. Statistical analyses of the data were performed using STATISTICA software, version 6.1 (StatSoft, USA) and R, version 2.15.1.

### Analysis of fecundity and fertility

Before analysis, control and experimental females of different ages were maintained with young males for mating during 24 h. Mated females were put separately into the vials with a medium colored with activated carbon for egg-laying for 24 h. To estimate the age-dependent changes of fecundity and fertility, one time a week the number of eggs laid by females during 24 h was calculated (fecundity), and the number of adult flies developed from these eggs was estimated after 10–15 days (fertility). A total of 50 females were analyzed per experimental variant. To compare statistical significance between control and experimental flies, t-Student test was used.

### Detection of intestinal permeability

As previously shown by Rera et al. the intestinal permeability can be used as a physiological marker of *Drosophila* aging  [11]. The intestinal barrier function was assessed in 10 weeks old flies by Smurf assay [11]. Control and experimental animals were maintained on dyed medium for 16 h. Dyed medium was prepared by adding 2.5% (wt/vol) of Blue dye No. 1 (Sigma Aldrich, USA) to yeast medium. The flies with increased intestinal permeability were blue-colored outside of the digestive tract and counted as Smurfs [10]. To estimate the statistical significance of differences, the Fisher’s exact test was used.

### Analysis of locomotor activity and sleep/rest parameters

The age-dependent dynamics of spontaneous locomotor activity and sleep was analyzed in 1-, 4-, and 6-week-old flies using the DAM5 *Drosophila* Locomotor Activity Monitor (Trikinetics, USA). Activity of was recorded in a 12 h:12 h light-dark cycle. Single flies were housed in 5 mm glass tubes containing 5% sucrose and 2% agarose medium. The 16 flies were analyzed per experimental variant in 3 replicates. For the locomotor activity analysis data from individual flies were collected during 72 h and represented as average total daily locomotor activity. For measuring sleep/rest parameters, locomotor activity data were collected in 1 min bins and analyzed for periods of ≥5 min with no activity. Currently, sleep/rest in *Drosophila* is defined as five contiguous minutes of complete inactivity [12]. The percent of time that flies spend sleeping/resting at one hour intervals during 24 h cycle was calculated [13].

### Stress resistance analysis

To assess the influence of fucoxanthin on the age-related changes in stress resistance, 150 flies (30 individuals per *Drosophila* narrow vial) were collected in each experimental variant at 9 different ages (every 7 days from the age of 7 days to 63 days). The following stressors were used: paraquat (oxidative stress), starvation, and hyperthermia. At the variant of hyperthermia, flies were kept in standard test vials on agar-yeast medium at 35 °C. For starvation flies were placed in vials with 5 ml of 1% agar solution. Before as exposed to paraquat flies were deprived of food and water for 3 h and then transferred into vials containing filter paper moistened with 300 ml of a 5% sucrose solution with 20 mM paraquat. Flies were transferred into new vials every two days, and kept under stress until the death.

Mortality was analyzed two times a day, separately for males and females. The mean, median survival time, and the time of 90% mortality were calculated. To compare the statistical differences in median lifespan between control and experimental groups, the Gehan-Breslow-Wilcoxon test was used [9]. A Wang-Allison test was used to estimate the differences in the age of 90% mortality [10]. Student’s t-test was used for evaluation of the significance of the difference in the slope of the two regression lines for the experiment and control. Statistical analysis of the data were performed using R, version 2.15.1 and online application for survival analysis - OASIS [14, 15].

### Total RNA extraction and qualification

Transcriptomic analysis was performed using control and experimental flies at the age of 2 (young), 4 (mature) and 6 weeks (old). Fifty males and females were prepared separately for each experimental variant in 3 replicates. Flies were collected, immediately snap frozen in liquid nitrogen and stored at − 80 °C. Total RNA was isolated from 30 flies (10 flies per replicate) using QIAzol Lysis Reagent (Qiagen, Netherlands) with the isopropanol precipitation. The concentration of RNA was assessed using Qubit®2.0 Fluorometer (Invitrogen, USA) and NanoDrop® ND-1000 spectrophotometer (NanoDrop Technologies Inc., USA). The A260/A280 ratios of RNA samples were 1.8–2.0. All samples were treated with DNase I (Promega, USA).

### Library preparation and sequencing of mRNA

Double stranded cDNA library was prepared by using NEBNext® Ultra™ Directional RNA Library Prep kit for Illumina following manufacturer’s protocol from 1 mg of total RNA. Fragmentation was carried out using divalent cations under elevated temperature in NEBNext First Strand Synthesis Reaction Buffer (5X). First strand cDNA was synthesized using random hexamer primer and ProtoScript II Reverse Transcriptase. Second strand cDNA synthesis was subsequently performed using Second Strand Synthesis Enzyme Mix. The ds cDNA was isolate from the second-strand reaction mix using Ampure XP beads. The end-repair reaction was used to create blunt ends on the ds cDNA. To avoid the ligation of blunt ends during the adapter ligation reaction, a single ‘A’ nucleotide was added to the 3′ ends of them. The specific RNA Adapter Indexes supplied in the kit were ligated to cDNA fragments. The PCR process (15 cycles) was used to selectively enrich DNA fragments with adapter molecules on both ends and to amplify the amount of DNA in the library, according to the manufacturer’s protocol. Finally, the PCR products were purified (AMPure XP System). The quantity of libraries was determined using the qPCR method by Rotor-Gene 6000 PCR System (Qiagen, USA) according to the manufacturer’s protocol. Primers matched sequences within adapters flanking an Illumina sequencing library. Before starting qPCR, a control template was selected to measure the libraries for quantification. The quality of the libraries was checked on Agilent 2100 Bioanalyzer using a High Sensitivity DNA chip (Agilent Technologies, USA). The final library products were bands at approximately 260 bp. cDNA libraries were normalized to 4 nM, pooled together in equal volumes, and sequenced with 75 bp single-end reads on the NextSeq500 System (Illumina, USA). The sequencing data were stored in FASTQ format. At least 20 million reads were obtained for each sample.

### RNA sequencing data analysis

The initial processing of data from the device in the format fastq.gz was made by the program Kallisto [16]. At the output of which RNA expression values were obtained for each sample in estimated counts and TPMs (transcripts per million). To detect the differentially expressed (DE) genes, Bioconductor/R-project package (R version 3.3.1) was used with DESeq2. The KEGG (Kyoto Encyclopedia of Genes and Genomes) Pathways database was used to find pathways in which the differentially expressed genes are involved [17]. The GSEA (gene set enrichment analysis) analysis was performed using the software package R GSEAbase of Bioconductor [18].

## Results

### Lifespan

**Fig. 1:**
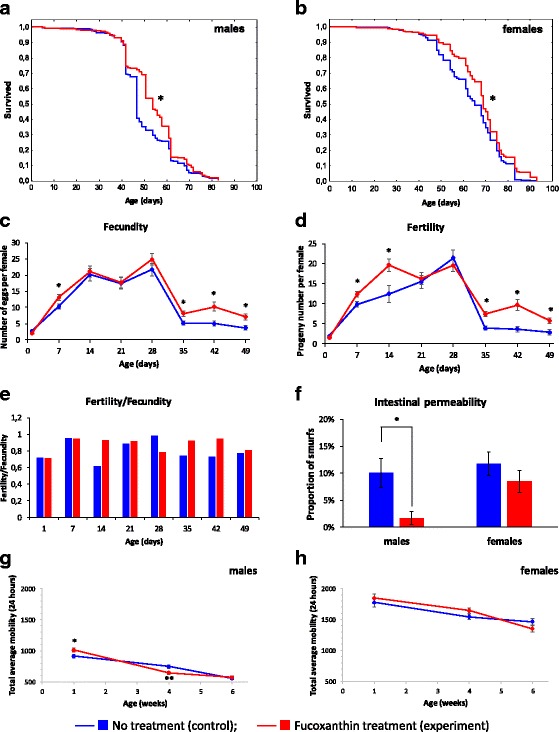
Influence of fucoxanthin on the Lifespan of males (**a**) and females (**b**). The results of 2 replications are combined. *p < 0.001, Kolmogorov-Smirnov test; Age-dependent dynamics of fecundity (**c**), fertility (**d**), and the ratio between fertility and fecundity (**e**). The error bars show standard errors. *p < 0.05, Student’s t-test; Intestinal permeability in the Smurf assay (**f**). The error bars show standard error of the proportion. *p < 0.01, Fisher’s exact test; Age-dependent dynamics of total daily locomotor activity of males (**g**) and females (**h**). The error bars show standard errors. *p < 0.05 **p < 0.01, Student’s t-test

In this study, we reproduced the lifespan extending effect of fucoxanthin in *Drosophila*, which was published earlier [7]. Treatment with 1 μM of fucoxanthin led to an increase in median lifespan of *Drosophila* males (by 14.9%, *p* < 0.001) (Table 1, Fig. 1a) and females (by 6.2%, p < 0.001) (Table 1, Fig. 1b) compared with the untreated control. At the same time, fucoxanthin decreased MRDT in males, and to a lesser extent in females. The decreasing of MRDT suggests that geroprotective effect of fucoxanthin in *Drosophila* is associated with prolongation of healthspan.

**Table 1:** Influence of fucoxanthin on median and maximum lifespan (the results of 2 replicates are combined)

Variant	Sex	M (days)	dM (%)	90% (days)	d90% (%)	MRDT (days)	dMRDT (%)	n
No treatment (control)	males	47		68		9.25		319
Fucoxanthin treatment (experiment)	males	54*	14.9	71	4.4	8.51	−8	319
No treatment (control)	females	65		83		7.99		323
Fucoxanthin treatment (experiment)	females	69*	6.2	83	0	7.94	−0.6	315

*M* - Median lifespan; 90% - age of 90% mortality (maximum lifespan); *MRDT* - Mortality rate doubling time; *dM, d90%*, *dMRDT* - Differences between median lifespan, age of 90% mortality, and MRDT of control and experimental flies, respectively; *n* - Number of flies; *p < 0.001, Gehan-Breslow-Wilcoxon test

The aging process and physiological health are interrelated with reproduction [19], motion ability [20], sleep/rest parameters [21], and intestinal integrity [22] in *Drosophila melanogaster* and other species. Therefore, we investigated the effect of fucoxanthin on these parameters of the physiological state and health.

### Fecundity and fertility

We have shown the positive effect of fucoxanthin treatment on fecundity of females at the age of 7, 35, 42 and 49 days (by 26%, 55%, 105% and 92%, respectively, *p* < 0.05) (Fig. 1c) and fertility at the age of 7, 14, 35 and 42 days (by 25%, 57%, 92%, and 164%, respectively, p < 0.05) (Fig. 1d). Also, in females treated with fucoxanthin, a significant increase in the ratio between fertility and fecundity was observed at the age of 14, 35, and 42 days. However, at the age of 28 days the percentage of progeny developed to imago in fucoxanthin treated flies significantly decreased (Fig. 1e). Recently, Lashmanova and coauthors also observed the stimulating effect of 1 μM fucoxanthin on fertility [7].

### Intestinal permeability

The proportion of flies with increased intestinal permeability (Smurfs) in the experimental group of males (1.7%) is more than 5 times less than in the control group (10.1%) (Fig. 1f). In females, the differences between experiment and control variants were not observed. It was recently shown that intestinal permeability is physiological marker of aging conserved across a broad range of invertebrate and vertebrate species including 3 *Drosophila* species (*D. melanogaster, D. mojavensis,* and *D. virilis*)*,* nematode *Caenorhabditis elegans* as well as zebrafish *Danio rerio* [10, 22]. Previously, Rera et al. showed that lifespan extending interventions in *Drosophila* can mitigate aging-related changes in the intestine [10, 23]. The results of this assay are consistent with the increase of median lifespan (*p* < 0.001) of males from the experimental group (54 days) in comparison with males from the control group (47 days) (Table 1, Fig. 1a). Despite the geroprotective effect of fucoxanthin on individuals of both sexes (Table 1, Fig. 1), a significant improvement in intestinal barrier function was observed in males only. Because the increase of intestinal permeability followed by a high risk of death [22] the obtained results may reflect longer lifespan of females in comparison with males.

### Locomotor activity

The dynamics of the age-dependent changes of locomotor activity was estimated in flies at the age of 1, 4, 6 weeks. It was found that fucoxanthin increased the total daily activity of young males (by 10%) and decreased the activity of mature males (by 13%) (Fig. 1g). At the same time, the activity of old males and all-age females did not change (Fig. 1h). According to Lashmanova and coauthors, fucoxanthin increases the locomotor activity of males, but does not affect the activity of females [7].

**Fig. 2:**
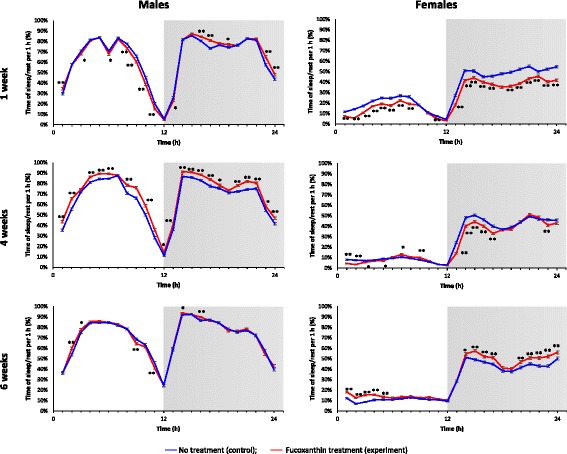
Effect of fucoxanthin on age-dependent dynamics of sleep/rest parameters. The white and gray background colors indicate a 12 h:12 h light-dark cycle, respectively. The error bars show standard error of the proportion. *p < 0.05, **p < 0.01, Fisher’s exact test

We also found that fucoxanthin increases the amount of sleep/rest periods during the night in males at all ages and in old females. At the same time fucoxanthin decreases the amount of sleep/rest periods during the day in young males and females, but increases it in mature males and old females (Fig. 2). Aging of model organisms and human is associated with loss of sleep consolidation when the sleep time increases in the daytime, but decreases during the night [11, 21]. Therefore, overcoming of age-related sleep disturbances will lead to an improvement of the quality of life for older people.

### Stress resistance

**Fig. 3:**
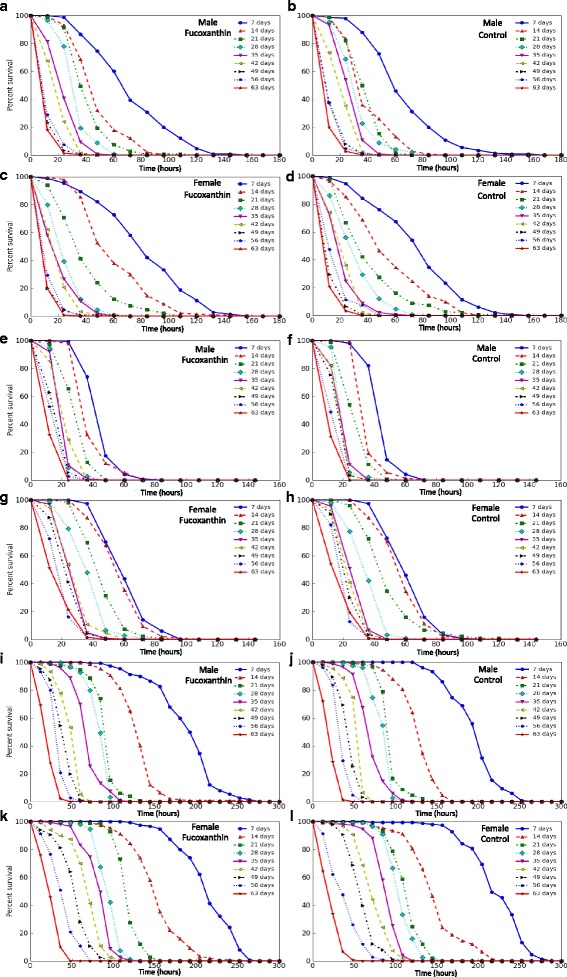
Influence of fucoxanthin on *Drosophila melanogaster* stress-resistance in different ages. Exposure to paraquat (**a, b, c, d**); Exposure to starvation (**e, f, g, h**); Exposure to hyperthermia (**i, j, k, l)**

The influence of fucoxanthin on survival rates of flies under conditions of oxidative stress (paraquat), proteotoxic stress (hyperthermia) and starvation were estimated. In the flies of both sexes from experimental and control groups, we observed age-related changes in shape and compression of the survival curves under the influence of all the used stress factors (Fig. 3).

In order to compare the aging-related dynamics of survival of flies treated with the fucoxanthin and control flies, the regression lines scatterplots were built based on the mean survival time for all experimental variants at each age group (Additional file 1). Both control and experimental groups, have a high negative correlation between stress resistance and age. The observed effect may be explained by the age-related decrease in stress-resistance and physiological functions [24, 25]. However, in all variants, no significant differences in the age-dependent dynamics were found based on comparing angle of inclination of regression lines scatterplots of experimental and control groups (*p* > 0.05, Student’s t-test). Despite fucoxanthin changed (decrease or increase) the median survival and the time of 90% mortality of the flies in stress conditions at few variants, these effects are not regular and have a stochastic character (Additional file 2). Thus we can conclude that fucoxanthin did not affect age-related decline of stress resistance in *Drosophila*.

Although in previous studies fucoxanthin demonstrated an antioxidant activity [26] and increased the resistance of *Drosophila* to oxidative stress [7], in our study it did not protect the flies from deleterious action of paraquat. We also did not find the effect of fucoxanthin on the resistance of flies to starvation and hyperthermia, which is in accordance with the previously published results [7].

### Transcriptome analysis

**Fig. 4:**
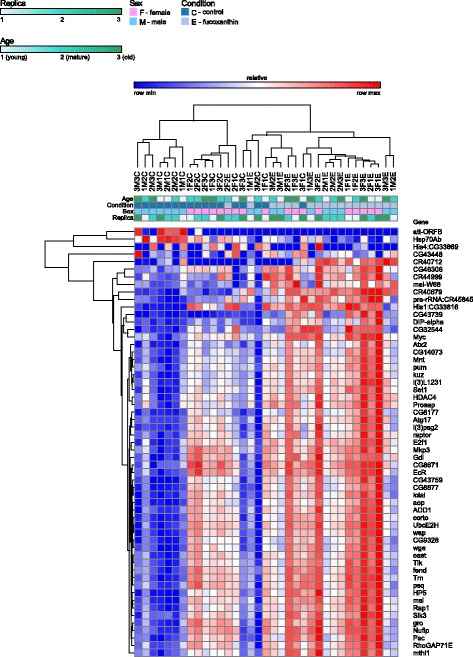
Differential expression of genes, involved in response to the fucoxanthin treatment

We derived RNA sequencing expression profiles for 12,000 genes (after eliminating low expression ones) (Additional file 3). As a result of the analysis of differential expression of genes, the top 57 genes, authentically involved in response to the fucoxanthin treatment were identified (Fig. 4). It is noteworthy that in the old flies fucoxanthin alters the expression level of a much larger number of genes than in the young flies (Additional file 4). When the selected threshold of expression was 2-fold or more (FDR < 0.05) in flies at the age of 2 weeks, fucoxanthin changed expression of 3 genes, in flies at the age of 4 weeks - 10 genes, whereas in flies at the age of 6 weeks, the expression level of 49 genes were affected.

Fucoxanthin treatment of young flies down-regulated *att-ORFB*, *Hsp70Ab*, and *His4:CG33869* genes expression (Additional file 4). The heat shock protein (*hsp*) genes are involved in biological processes such as responses to unfolded proteins, hypoxia, heat, etc. The *hsp* genes have been shown to regulate both lifespan and resistance of organism to adverse environmental factors [27, 28]. It is known that the heat shock proteins may be induced by oxidative stress through the JNK signaling pathway and the transcription factor Foxo [27]. The fucoxanthin considered to be a potential antioxidant [26]. In addition, in young male flies fucoxanthin activates mRNA expression of antioxidant enzyme sod3 (Additional file 4). Thus fucoxanthin may be resposible for down-regulation of *hsp70* gene through activation of antioxidant gene expression as well as by free radical scavenging. Histone H4, one of the four histones that, along with H3, plays a central role in nucleosome formation and is involved in the assembly of nucleosome, DNA-templated transcription, initiation. In yeast, aging is accompanied by a loss of histone proteins from the genome, but increasing the histone supply by inactivation of the Hir (histone information regulator) complex or overexpression of histones extends lifespan [29]. Therefore, observed down-regulation of *His4:CG33869* gene is not fully consistent with the geroprotective properties of fucoxanthin.

Fucoxanthin treatment of mature flies down-regulated male-specific *att-ORFB* gene and up-regulated *mthl1*, *CR40679*, *mei-W68*, *CR40712*, *DIP-alpha*, *CR45845*, *CG43739*, *CG43448*, and *CG46306* genes (Additional file 4). The *mthl1* gene is a member of *methuselah* gene family of G-protein coupled receptors [30]. A recent genome-wide association study identifed association of *mthl1* with microbiota-dependent nutritional effects [31]. Another members of *methuselah* gene family are involved in the control of stress resistance, lifespan and healthspan in *Drosophila*. For example, the *mthl3*, *mthl-9*, and *mthl-11* were downregulated by both endurance training and selective breeding for longevity [32]. In addition, the *mth* gene plays an important role in lifespan regulation and resistance to various forms of stress [33, 34]. Paradoxically, the overexpression of *mth* targeted to the same cells has similar pro-longevity effects to reduced expression due to interaction of methuselah with β-arrestin, which uncouples G-protein-coupled receptors from their G-proteins [34].

In old flies fucoxanthin up-regulated 49 genes (Additional file 4). There are genes involved in MAPK signaling pathway, such as *СG3166*, *CG8384*; in Wnt signaling pathway, such as *СG10798*, *CG8384*; in autophagy, such as *СG1347*, *CG4320*. In addition, *CG4320* (also known as *dRaptor*) is involved in longevity-regulating pathway and mTOR signaling pathway [35]. Also worthy of attention are the following genes: *mthl1* (see above), *ataxin-2*, *Mkp3* and *E2F1*. Recent studies have shown that *ataxin-2* deficiency correlates with insulin resistance and dyslipidemia, an action mediated via the mTOR pathway, suggesting that *ataxin-2* might play key roles in metabolic homeostasis including body weight regulation, insulin sensitivity, and cellular stress responses [36]. Also, *ataxin-2* is involved in some other biological process, such as neurogenesis and sleep. *Mkp3* gene mediates adult midgut epithelial homeostasis in *Drosophila*. *Mkp3* overexpression suppressed the growth of *Drosophila* intestinal stem cell tumors [37]. *E2F1* regulates the cell cycle progression and the response to DNA damage by post-translational modifications [38].

Fucoxanthin has garnered much attention for its anti-obesity and anti-diabetic effects. It have been shown to impact the lipid metabolism, adiposity, and related conditions in mammals [6]. Its anti-obesity effects in rodent models are apparently mediated by downregulation of lipogenic enzymes, upregulation of lipolytic enzymes, and induction of the expression of uncoupling proteins. We found that in young and mature *Drosophila* males fucoxanthin downregulated expression of *Zw* gene that is homologue of mammalian lipogenic enzyme glucose-6-phosphate dehydrogenase (Additional file 5).

**Fig. 5:**
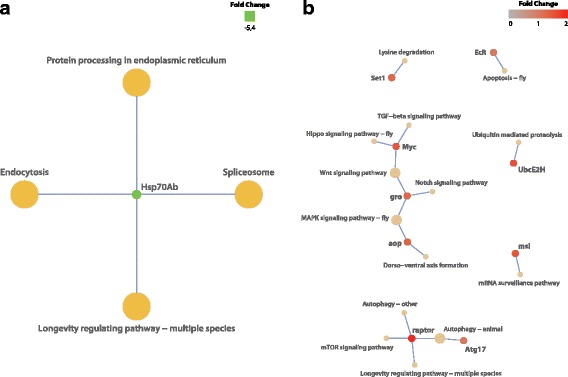
Down- and up-regulated KEGG pathways and genes representing it under fucoxanthin diet in young (**a**) and old (**b**) flies of both sexes

KEGG database contains systematic analysis of inner-cell metabolic pathways and functions of gene products. It helps studying complicated biological behaviors of genes. We identified 57 DE genes involved in 17 KEGG pathways (Figs. 4 and 5). In young flies fucoxanthin inhibited molecular pathways related to endocytosis, protein processing in endoplasmic reticulum, spliceosome, and longevity regulating (Fig. 5a, Additional files 6 and 7). The most represented molecular pathways induced by fucoxanthin in old flies is related to longevity, including MAPK, mTOR, Wnt, Notch, and Hippo signaling pathways, аutophagy, apoptosis (Fig. 5b, Additional files 6 and 7). Thus transcriptome analysis revealed age-related increase in the number of up-regulated KEGG pathways and genes representing it under fucoxanthin diet.

## Discussion

Transcriptome analysis can elucidate the molecular mechanisms of the fucoxanthin at the whole organism level. Despite the lifespan-extending effect of fucoxanthin was not sex-specific, in males it induced the differential expression of a larger number of genes than in females (Table 2, and Additional file 5). In males, the fucoxanthin affects the expression of genes involved in the signaling pathways such as DNA replication, Metabolic pathways, Oxidative phosphorylation, Purimidine metabolism, Purine metabolism, Base excision repair, Nucleotide excision repair. The stress response, including purimidine and purine metabolism, was found to be up-regulated during aging [39]. Impairment of oxidative phosphorylation during aging is well known for several model organisms [40].

**Table 2:** Influence of fucoxanthin on differential expression of genes in males and females

Age	Number of DE genes (total (up-regulated/down-regulated))	
	males	females
2 weeks	1584 (1120/464)	11 (7/4)
4 weeks	1134 (218/916)	46 (39/7)
6 weeks	321 (14/307)	69 (48/21)

It is known, that the genes that differ in expression between the sexes are known as sex-biased genes and can be classified as either male-biased or female-biased, depending on which sex has the higher expression [41]. In *Drosophila melanogaster*, 15–70% of known genes have sexually dimorphic expression [42, 43]. There have been several previous attempts to connect changes in gene expression to diet manipulation in *Drosophila* [44–46]. Wyman et al. showed that in *Drosophila*, males and females that are provided with a high-quality diet exhibit greater sex-biased expression than those fed a low-quality diet [43]. There is no evidence that X-linked CDSD (condition-dependent sexual dimorphism) is stronger or weaker than autosomal CDSD in their study. However, female-biased genes expressed in the female-soma have the lowest degree of CDSD compared to the other gene categories. More data support the idea that mating in *Drosophila melanogaster* induces a series of changes in the female’s behavior and physiology, including decreasing her attractiveness to other males, decreasing her receptivity to future matings, increasing her food consumption, elevating her egg-laying rate, facilitating storage and utilization of sperm, and decreasing her life span. McGraw et al. demonstrated that there is essential alteration of post-mating gene expression profiles of female *Drosophila melanogaster* [45]. Thus the observed differences in number of DE genes between males and females (Table 2, Additional file 5) are apparently related with sexually dimorphic expression in response to fucoxanthin treatment. The sex-biased expression profile can vary greatly among tissues and lead to sex-related differences in the effects of fucoxanthin in the various levels of biological organization.

We also found that fucoxanthin significantly improved aging-associated intestinal barrier function in old males. Transcriptome analysis revealed that fucoxanthin down-regulate the expression of antimicrobial peptide gene *DptB* (*Diptericin B*) in middle age males (Additional file 5). The age-related increase in expression of antimicrobial peptide genes (*drosomycin* (*Drs*), *drosocin* (*Dro*), and *diptericin* (*Dpt*)) is tightly linked to intestinal barrier dysfunction [10].

According to our results, fucoxanthin can attenuate sleep disturbance in old individuals at night. RNA sequencing showed that fucoxanthin causes an increase in the expression level of the *ataxin-2* gene (Fig. 4) which is involved in sleep regulation in *Drosophila* [47].

In our study fucoxanthin did not protect the flies from deleterious action of paraquat, starvation and hyperthermia. According to RNA sequencing data, fucoxanthin down-regulated *hsp70* in young flies, but did not cause significant changes in the level of transcription of stress resistance genes.

## Conclusion

In this paper, we examined the effects of fucoxanthin on the *Drosophila* aging process at the molecular and the whole organism levels. At the organismal level, fucoxanthin increased the median lifespan and had a positive effect on fecundity, fertility, intestinal barrier function, and nighttime sleep. Total RNA sequencing revealed that the fucoxanthin treatment influenced the expression of genes involved in a variety of cellular processes including longevity regulating pathway, autophagy, translation, glycolysis, oxidative phosphorylation, apoptosis, immune response, neurogenesis, sleep, and response to DNA damage. Among the most represented molecular pathways induced by fucoxanthin treatment, a significant portion is related to longevity. Thus, the effects of fucoxanthin at the whole organism is associated with differential expression of genes and changes in the activity of molecular pathways.

Recent findings suggest that geroprotective effects of different dietary phytochemicals is associated with hormesis [48–50]. Activation of cellular defense mechanisms in response to mild phytochemicals stress may leads to beneficial effects on healthspan and longevity [51–54]. Hormesis associated with activation of kinases and transcription factors that induce the expression of genes that encode antioxidant enzymes, DNA repair proteins, immune response proteins, protein chaperones, and other cytoprotective factors [28, 49, 55, 56]. In transcriptome analysis of the effects of fucoxanthin treatment we revealed activation of the mechanisms related to hormesis. Thus hormesis may be considered as one of the probable mechanisms of geroprotective effects of fucoxanthin.

## Additional files

Additional file 1: The scattering diagrams showing the dependence of survival on the age of *D. melanogaster* treated with fucoxanthin and control groups under the impact of various stress factors: paraquat (a – males; b – females), starvation (c – males; d – females), hyperthermia (e – males; f – females); color lines - linear regression. (PDF 89 kb)

Additional file 2: The influence of fucoxanthine on the *Drosophila* survival in different stress conditions. (XLSX 15 kb)

Additional file 3: Gene expression heat map. (PDF 5909 kb)

Additional file 4: Differentially expressed genes in response to the fucoxanthin treatment. (XLSX 15 kb)

Additional file 5: Influence of fucoxanthin on differential expression of genes in males and females in different ages. (XLSX 190 kb)

Additional file 6: Significant molecular pathways induced by fucoxanthin. (PDF 643 kb)

Additional file 7: Significant molecular pathways induced by fucoxanthin in flies with different ages. (XLSX 12 kb)

## Abbreviations

Bp: base pairs
DE: differentially expressed
GSEA: gene set enrichment analysis
Hsp: heat shock protein
KEGG: Kyoto Encyclopedia of Genes and Genomes
LD: light-dark
MRDT: mortality rate doubling time
rRNA: ribosomal RNA
TPMs: transcripts per million

## References

[1] Peng J, Yuan JP, Wu CF, Wang JH (2011). Fucoxanthin, a marine carotenoid present in brown seaweeds and diatoms: metabolism and bioactivities relevant to human health. Mar Drugs..

[2] Kelman D, Posner EK, McDermid KJ, Tabandera NK, Wright PR, Wright AD (2012). Antioxidant activity of Hawaiian marine algae. Mar Drugs..

[3] Raposo MF, de Morais AM, de Morais RM (2015). Carotenoids from marine microalgae: a valuable natural source for the prevention of chronic diseases. Mar Drugs.

[4] Dembitsky VM, Maoka T (2007). Allenic and cumulenic lipids. Prog Lipid Res.

[5] D'Orazio N, Gemello E, Gammone MA, de Girolamo M, Ficoneri C, Riccioni G (2012). Fucoxantin: a treasure from the sea. Mar Drugs..

[6] Muradian K, Vaiserman A, Min KJ, Fraifeld VE (2015). Fucoxanthin and lipid metabolism: a minireview. Nutr Metab Cardiovasc Dis.

[7] Lashmanova E, Proshkina E, Zhikrivetskaya S, Shevchenko O, Marusich E, Leonov S, Melerzanov A, Zhavoronkov A, Moskalev A (2015). Fucoxanthin increases lifespan of *Drosophila melanogaster* and *Caenorhabditis elegans*. Pharmacol Res.

[8] Fleming TR, O'Fallon JR, O'Brien PC, Harrington DP (1980). Modified Kolmogorov-Smirnov test procedures with application to arbitrarily right-censored data. Biometrics.

[9] Breslow N. A generalized Kruskal-Wallis test for comparing K samples subject to unequal patterns of censorship. Biometrika. 1970579-94.

[10] Wang C, Li Q, Redden DT, Weindruch R, Allison DB. Statistical methods for testing effects on "maximum lifespan". Mechanisms of ageing and development. 2004;125:629-32. 10.1016/j.mad.2004.07.003 15491681

[11] Rera M, Clark RI, Walker DW. Intestinal barrier dysfunction links metabolic and inflammatory markers of aging to death in *Drosophila*. Proc Natl Acad Sci USA. 2012;109:21528-33. 10.1073/pnas.1215849110 PMC3535647 23236133

[12] Shaw PJ, Cirelli C, Greenspan RJ, Tononi G. Correlates of sleep and waking in *Drosophila melanogaster*. Science. 2000;287:1834-7. 10.1126/science.287.5459.1834 10710313

[13] Chiu JC, Low KH, Pike DH, Yildirim E, Edery I. Assaying locomotor activity to study circadian rhythms and sleep parameters in *Drosophila*. J Vis Exp. 2010;43:e2157. 10.3791/2157 PMC3229366 20972399

[14] Yang J-S, Nam H-J, Seo M, Han SK, Choi Y, Nam HG, Lee S-J, Kim S. OASIS: Online application for the survival analysis of lifespan assays performed in aging research. PLoS One. 2011;6:e23525. 10.1371/journal.pone.0023525 PMC3156233 21858155

[15] R Core Team. R: A language and environment for statistical computing. R Foundation for Statistical Computing, Vienna, Austria. http://www.R-project.org/. 2013.

[16] Bray NL, Pimentel H, Melsted P, Pachter L (2016). Near-optimal probabilistic RNA-seq quantification. Nat Biotech.

[17] Kanehisa M, Furumichi M, Tanabe M, Sato Y, Morishima K (2017). KEGG: new perspectives on genomes, pathways, diseases and drugs. Nucleic Acids Res.

[18] Luo W, Brouwer C (2013). Pathview: an R/Bioconductor package for pathway-based data integration and visualization. Bioinformatics.

[19] Rogina B, Wolverton T, Bross TG, Chen K, Muller HG, Carey JR (2007). Distinct biological epochs in the reproductive life of female *Drosophila melanogaster*. Mech Ageing Dev.

[20] Le Bourg E, Lints FA (1984). A longitudinal study of the effects of age on spontaneous locomotor activity in *Drosophila melanogaster*. Gerontology.

[21] Koh K, Evans JM, Hendricks JC, Sehgal A (2006). A *Drosophila* model for age-associated changes in sleep:wake cycles. Proc Natl Acad Sci U S A.

[22] Dambroise E, Monnier L, Ruisheng L, Aguilaniu H, Joly JS, Tricoire H, Rera M (2016). Two phases of aging separated by the Smurf transition as a public path to death. Sci Rep.

[23] Rera M, Bahadorani S, Cho J, Koehler CL, Ulgherait M, Hur JH, Ansari WS, Lo T, Jones DL, Walker DW (2011). Modulation of longevity and tissue homeostasis by the *Drosophila* PGC-1 homolog. Cell Metab.

[24] Kregel KC (2002). Invited review: heat shock proteins: modifying factors in physiological stress responses and acquired thermotolerance. J Appl Physiol.

[25] López-Otín C, Blasco MA, Partridge L, Serrano M, Kroemer G (2013). The hallmarks of aging. Cell.

[26] Sangeetha RK, Bhaskar N, Baskaran V (2009). Comparative effects of β-carotene and fucoxanthin on retinol deficiency induced oxidative stress in rats. Mol Cell Biochem.

[27] Tower J. Heat shock proteins and *Drosophila* aging. Exp Gerontol 2011;46:355–362. 10.1016/j.exger.2010.09.002 PMC3018744 20840862

[28] Moskalev A, Shaposhnikov M, Turysheva E (2009). Life span alteration after irradiation in *Drosophila melanogaster* strains with mutations of *Hsf* and *Hsp*s. Biogerontology.

[29] Feser J, Truong D, Das C, Carson JJ, Kieft J, Harkness T, Tyler JK (2010). Elevated histone expression promotes life span extension. Mol Cell.

[30] Araújo AR, Reis M, Rocha H, Aguiar B, Morales-Hojas R, Macedo-Ribeiro S, Fonseca NA, Reboiro-Jato D, Reboiro-Jato M, Fdez-Riverola F, Vieira CP, Vieira J (2013). The *Drosophila melanogaster methuselah* gene: a novel gene with ancient functions. PLoS One.

[31] Dobson AJ, Chaston JM, Newell PD, Donahue L, Hermann SL, Sannino DR, Westmiller S, Wong AC, Clark AG, Lazzaro BP, Douglas AE (2015). Host genetic determinants of microbiota-dependent nutrition revealed by genome-wide analysis of *Drosophila melanogaster*. Nat Commun.

[32] Sujkowski A, Bazzell B, Carpenter K, Arking R, Wessells RJ (2015). Endurance exercise and selective breeding for longevity extend *Drosophila* healthspan by overlapping mechanisms. Aging (Albany NY).

[33] Lin YJ, Seroude L, Benzer S (1998). Extended life-span and stress resistance in the *Drosophila* mutant *methuselah*. Science.

[34] Gimenez LE, Ghildyal P, Fischer KE, Hu H, Ja WW, Eaton BA, Wu Y, Austad SN, Ranjan R (2013). Modulation of methuselah expression targeted to *Drosophila* insulin-producing cells extends life and enhances oxidative stress resistance. Aging Cell.

[35] Ni Q, Gu Y, Xie Y, Yin Q, Zhang H, Nie A, Li W, Wang Y, Ning G, Wang W, Wang Q (2017). Raptor regulates functional maturation of murine beta cells. Nat Commun.

[36] Carmo-Silva S, Nobrega C, Pereira de Almeida L, Cavadas C (2017). Unraveling the role of Ataxin-2 in metabolism. Trends Endocrinol Metab.

[37] Patel PH, Dutta D, Edgar BA (2015). Niche appropriation by *Drosophila* intestinal stem cell tumours. Nat Cell Biol.

[38] Glorian V, Allegre J, Berthelet J, Dumetier B, Boutanquoi PM, Droin N, Kayaci C, Cartier J, Gemble S, Marcion G, Gonzalez D, Boidot R, Garrido C, Michaud O, Solary E, Dubrez L (2017). DNA damage and S phase-dependent E2F1 stabilization requires the cIAP1 E3-ubiquitin ligase and is associated with K63-poly-ubiquitination on lysine 161/164 residues. Cell Death Dis.

[39] Landis GN, Abdueva D, Skvortsov D, Yang J, Rabin BE, Carrick J, Tavare S, Tower J (2004). Similar gene expression patterns characterize aging and oxidative stress in *Drosophila melanogaster*. Proc Natl Acad Sci U S A.

[40] Lesnefsky EJ, Hoppel CL (2006). Oxidative phosphorylation and aging. Ageing Res Rev.

[41] Grath S, Parsch J (2016). Sex-biased gene expression. Annu Rev Genet.

[42] Moskalev A, Shaposhnikov M, Snezhkina A, Kogan V, Plyusnina E, Peregudova D, Melnikova N, Uroshlev L, Mylnikov S, Dmitriev A, Plusnin S, Fedichev P, Kudryavtseva A (2014). Mining gene expression data for pollutants (dioxin, toluene, formaldehyde) and low dose of gamma-irradiation. PLoS One.

[43] Wyman MJ, Agrawal AF, Rowe L (2010). Condition-dependence of the sexually dimorphic transcriptome in *Drosophila melanogaster*. Evolution.

[44] Harbison ST, Chang S, Kamdar KP, Mackay TF (2005). Quantitative genomics of starvation stress resistance in *Drosophila*. Genome Biol.

[45] McGraw LA, Clark AG, Wolfner MF (2008). Post-mating gene expression profiles of female *Drosophila melanogaster* in response to time and to four male accessory gland proteins. Genetics.

[46] Zinke I, Schutz CS, Katzenberger JD, Bauer M, Pankratz MJ (2002). Nutrient control of gene expression in *Drosophila*: microarray analysis of starvation and sugar-dependent response. EMBO J.

[47] Thimgan MS, Seugnet L, Turk J, Shaw PJ (2015). Identification of genes associated with resilience/vulnerability to sleep deprivation and starvation in *Drosophila*. Sleep.

[48] Calabrese V, Cornelius C, Mancuso C, Pennisi G, Calafato S, Bellia F, Bates TE, Giuffrida Stella AM, Schapira T, Dinkova Kostova AT, Rizzarelli E (2008). Cellular stress response: a novel target for chemoprevention and nutritional neuroprotection in aging, neurodegenerative disorders and longevity. Neurochem Res.

[49] Son TG, Camandola S, Mattson MP (2008). Hormetic dietary phytochemicals. NeuroMolecular Med.

[50] Calabrese V, Cornelius C, Dinkova-Kostova AT, Iavicoli I, Di Paola R, Koverech A, Cuzzocrea S, Rizzarelli E, Calabrese EJ (2012). Cellular stress responses, hormetic phytochemicals and vitagenes in aging and longevity. Biochim Biophys Acta.

[51] Le Bourg E (2009). Hormesis, Aging and longevity. Biochim Biophys Acta.

[52] Minois N (2000). Longevity and Aging: beneficial effects of exposure to mild stress. Biogerontology.

[53] Rattan SI (2008). Hormesis in aging. Ageing Res Rev.

[54] Shaposhnikov M, Latkin D, Plyusnina E, Shilova L, Danilov A, Popov S, Zhavoronkov A, Ovodov Y, Moskalev A (2014). The effects of pectins on life span and stress resistance in *Drosophila melanogaster*. Biogerontology.

[55] Tatar M, Khazaeli AA, Curtsinger JW (1997). Chaperoning extended life. Nature.

[56] Yang P, He XQ, Peng L, Li AP, Wang XR, Zhou JW, Liu QZ (2007). The role of oxidative stress in hormesis induced by sodium arsenite in human embryo lung fibroblast (HELF) cellular proliferation model. J Toxicol Environ Health A.

