# Supplementary material for: Transcriptome analysis reveals mechanisms of geroprotective effects of fucoxanthin in Drosophila
Source: BMC Genomics. 2018 Feb 9;19(Suppl 3):77. doi: 10.1186/s12864-018-4471-x (PMC5836829; doi:10.1186/s12864-018-4471-x)
Supplement: Supplementary file 1 — The scattering diagrams showing the dependence of survival on the age of D. melanogaster treated with fucoxanthin and control groups under the impact of various stress factors: paraquat (a – males; b – females), starvation (c – males; d – females), hyperthermia (e – males; f – females); color lines - linear regression. (PDF 89 kb) [file 12864_2018_4471_MOESM1_ESM.pdf]

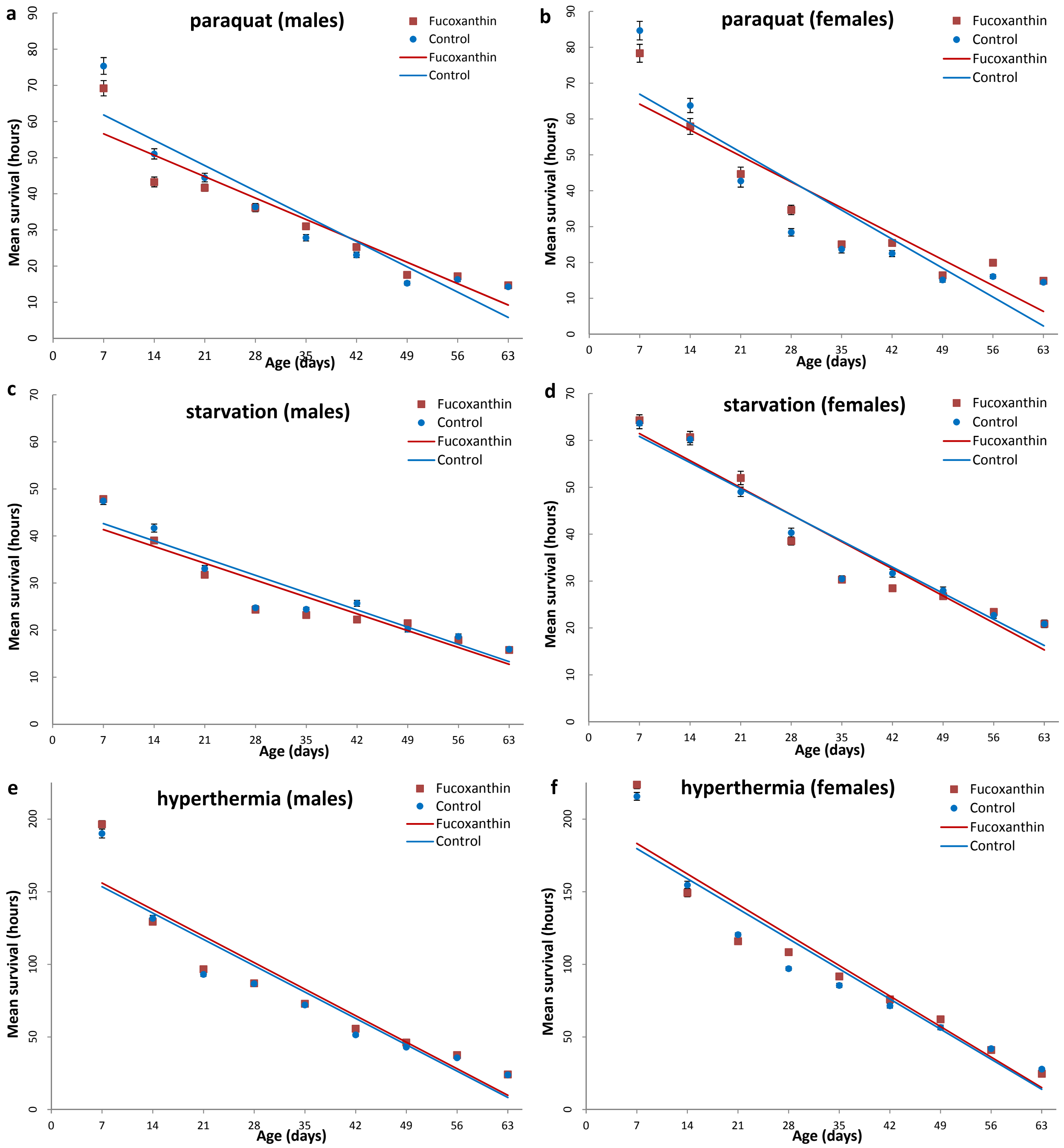

The scattering diagrams showing the dependence of survival on the age of *D. melanogaster* treated with fucoxanthin and control groups under the impact of various stress factors: paraquat (a – males; b – females), starvation (c – males; d – females), hyperthermia (e – males; f – females); color lines - linear regression
