# Supplementary figures and images for: Transcriptome analysis reveals mechanisms of geroprotective effects of fucoxanthin in Drosophila
Source: BMC Genomics. 2018 Feb 9;19(Suppl 3):77. doi: 10.1186/s12864-018-4471-x (PMC5836829; doi:10.1186/s12864-018-4471-x)

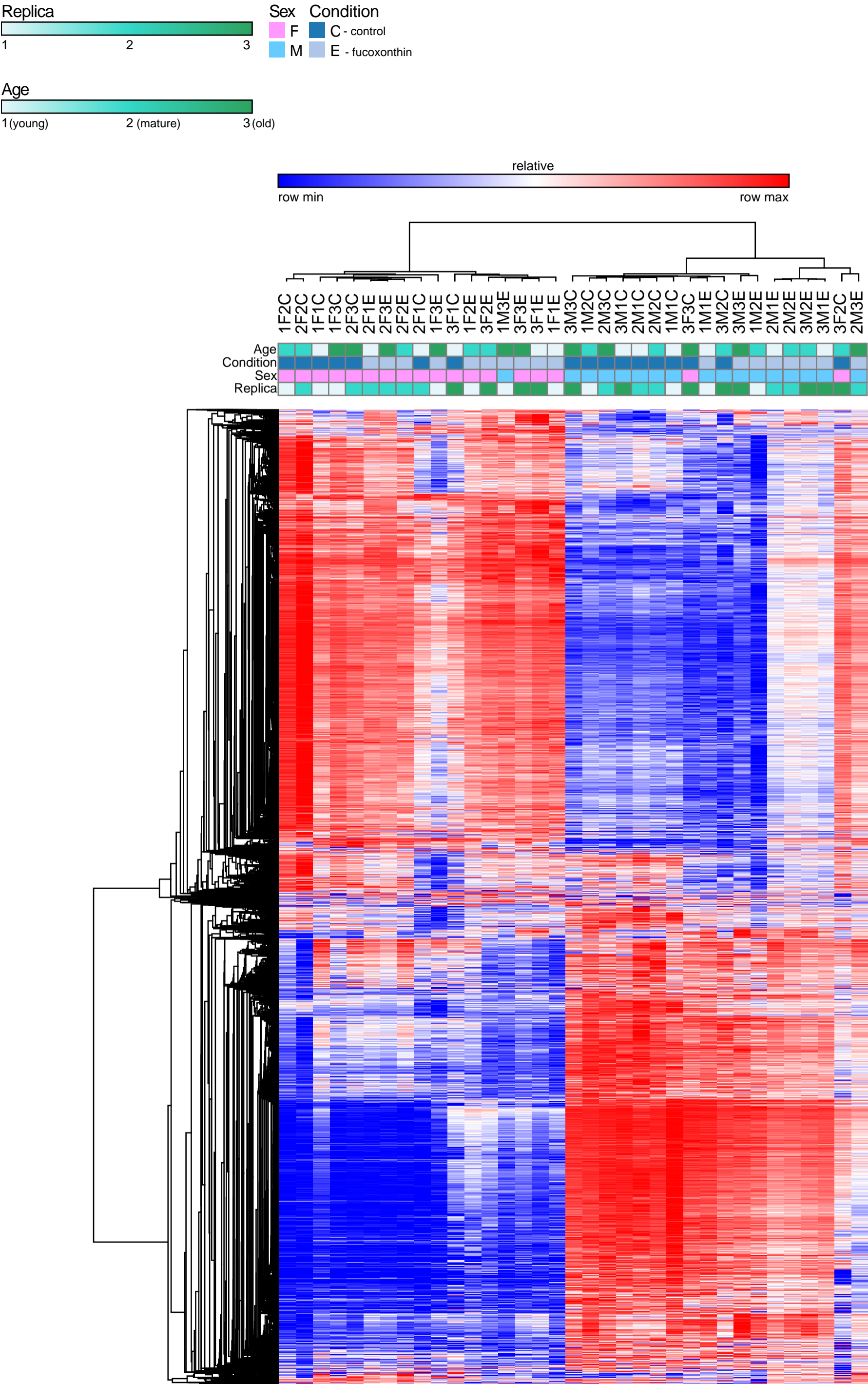

Supplement: Supplementary file 3 — Gene expression heat map. (PDF 5909 kb) [file 12864_2018_4471_MOESM3_ESM.pdf]
