# Supplementary material for: Transcriptome analysis reveals mechanisms of geroprotective effects of fucoxanthin in Drosophila
Source: BMC Genomics. 2018 Feb 9;19(Suppl 3):77. doi: 10.1186/s12864-018-4471-x (PMC5836829; doi:10.1186/s12864-018-4471-x)
Supplement: Supplementary file 6 — Significant molecular pathways induced by fucoxanthin. (PDF 643 kb) [file 12864_2018_4471_MOESM6_ESM.pdf]

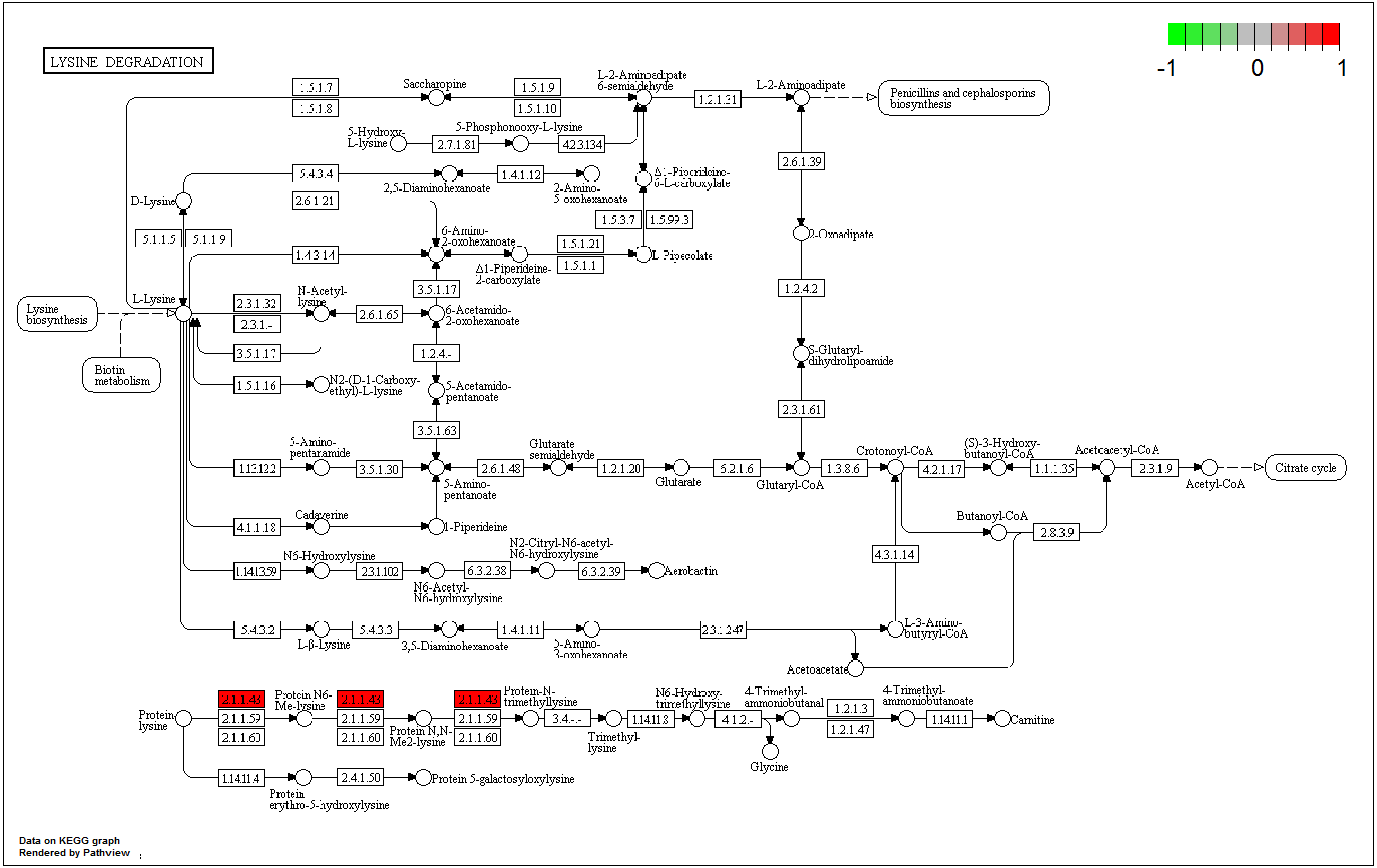

LYSINE DEGRADATION
-1
0
1
1.5.1.7
1.5.1.8
Saccharopine
1.5.1.9
1.5.1.10
L-2-Aminoadipate 6-semialdehyde
1.2.1.31
L-2-Aminoadipate
Penicillins and cephalosporins biosynthesis
5-Hydroxy-L-lysine
2.7.1.81
5-Phosphonooxy-L-lysine
4.2.3.134
5.4.3.4
1.4.1.12
2,5-Diaminohexanoate
2-Amino-5-oxohexanoate
Δ1-Piperideine-6-L-carboxylate
2.6.1.39
D-Lysine
2.6.1.21
1.5.3.7
1.5.99.3
5.1.1.5
5.1.1.9
6-Amino-2-oxohexanoate
1.5.1.21
1.5.1.1
L-Pipecolate
2-Oxoadipate
1.4.3.14
Δ1-Piperideine-2-carboxylate
3.5.1.17
1.2.4.2
Lysine biosynthesis
L-Lysine
2.3.1.32
2.3.1.-
N-Acetyl-lysine
2.6.1.65
6-Acetamido-2-oxohexanoate
S-Glutaryl-dihydrolipoamide
3.5.1.17
1.2.4.-
Biotin metabolism
1.5.1.16
N2-(D-1-Carboxy-ethyl)-L-lysine
5-Acetamido-pentanoate
2.3.1.61
3.5.1.63
5-Amino-pentanamide
Glutarate semialdehyde
Crotonoyl-CoA
(S)-3-Hydroxy-butanoyl-CoA
Acetoacetyl-CoA
1.13.12.2
3.5.1.30
2.6.1.48
1.2.1.20
Glutarate
6.2.1.6
Glutaryl-CoA
1.3.8.6
4.2.1.17
1.1.1.35
2.3.1.9
Acetyl-CoA
Citrate cycle
5-Amino-pentanoate
Butanoyl-CoA
4.1.1.18
Cadaverine
1-Piperideine
2.8.3.9
N2-Citryl-N6-acetyl-N6-hydroxylysine
N6-Hydroxylysine
4.3.1.14
1.14.13.59
2.3.1.102
6.3.2.38
6.3.2.39
Aerobactin
N6-Acetyl-N6-hydroxylysine
5.4.3.2
5.4.3.3
1.4.1.11
2.3.1.247
L-3-Amino-butyryl-CoA
L-β-Lysine
3,5-Diaminohexanoate
5-Amino-3-oxohexanoate
Acetoacetate
2.1.1.43
Protein N6-Me-lysine
2.1.1.43
2.1.1.43
Protein-N-trimethyllysine
N6-Hydroxy-trimethyllysine
4-Trimethyl-ammoniobutanal
4-Trimethyl-ammoniobutanoate
Protein lysine
2.1.1.59
2.1.1.59
2.1.1.59
3.4.-.-
1.14.11.8
4.1.2.-
1.2.1.3
1.14.11.1
Carnitine
2.1.1.60
2.1.1.60
2.1.1.60
Protein N,N-Me2-lysine
Trimethyl-lysine
1.2.1.47
Glycine
1.14.11.4
2.4.1.50
Protein 5-galactosyloxylysine
Protein erythro-5-hydroxylysine
Data on KEGG graph
Rendered by Pathview

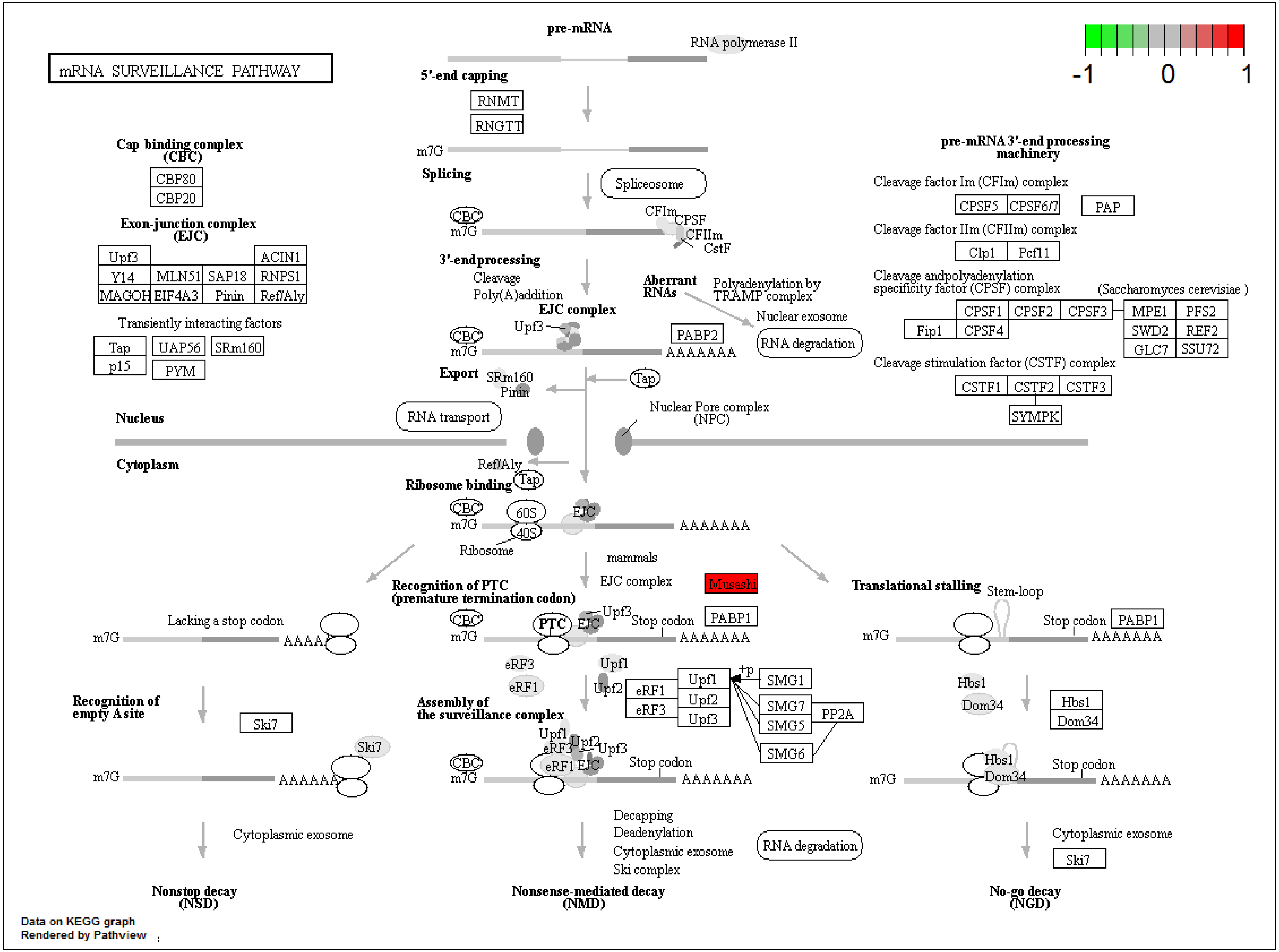

mRNA SURVEILLANCE PATHWAY
-1
0
1
pre-mRNA
RNA polymerase II
5'-end capping
RNMT
RNGTT
m7G
Splicing
Spliceosome
Cap binding complex (CBC)
CBP80
CBP20
Exon-junction complex (EJC)
Upf3
ACIN1
Y14
MLN51
SAP18
RNPS1
MAGOH
EIF4A3
Pinin
Ref/Aly
Transiently interacting factors
Tap
p15
UAP56
SRm160
PYM
CBC
CFIm
CPSF
CFIIm
CstF
3'-end processing
Cleavage
Poly(A)addition
EJC complex
Aberrant RNAs
Polyadenylation by TRAMP complex
Nuclear exosome
RNA degradation
PABP2
AAAAAAA
Export
SRm160
Pinin
RNA transport
Nuclear Pore complex (NPC)
Nucleus
Cytoplasm
pre-mRNA 3'-end processing machinery
Cleavage factor Im (CFIm) complex
CPSF5
CPSF6/7
PAP
Cleavage factor IIm (CFIIm) complex
Clp1
Pcf11
Cleavage and polyadenylation specificity factor (CPSF) complex
(Saccharomyces cerevisiae)
CPSF1
CPSF2
CPSF3
MPE1
PFS2
Fip1
CPSF4
SWD2
REF2
GLC7
SSU72
Cleavage stimulation factor (CSTF) complex
CSTF1
CSTF2
CSTF3
SYMPK
Ref/Aly
Ribosome binding
60S
40S
EJC
Ribosome
mammals
EJC complex
Musashi
Recognition of PTC (premature termination codon)
Translational stalling
Stem-loop
Lacking a stop codon
AAAA
PTC
Stop codon
PABP1
eRF3
eRF1
Upf1
Upf2
SMG1
+p
SMG7
SMG5
PP2A
SMG6
Hbs1
Dom34
Recognition of empty A site
Ski7
Assembly of the surveillance complex
AAAAAA
Cytoplasmic exosome
Decapping
Deadenylation
Ski complex
Nonstop decay (NSD)
Nonsense-mediated decay (NMD)
No-go decay (NGD)
Data on KEGG graph
Rendered by Pathview

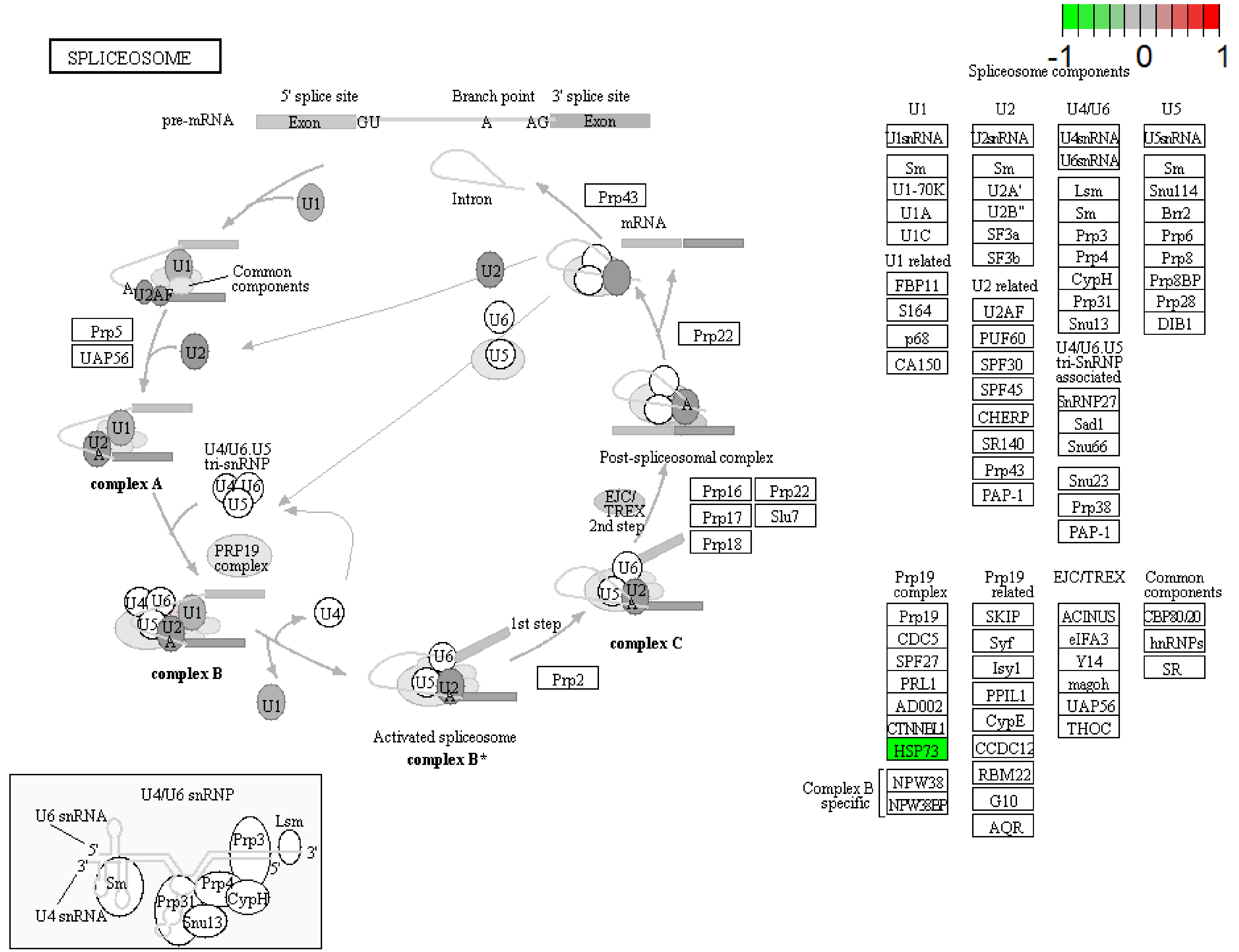

Data on KEGG graph
Rendered by Pathview

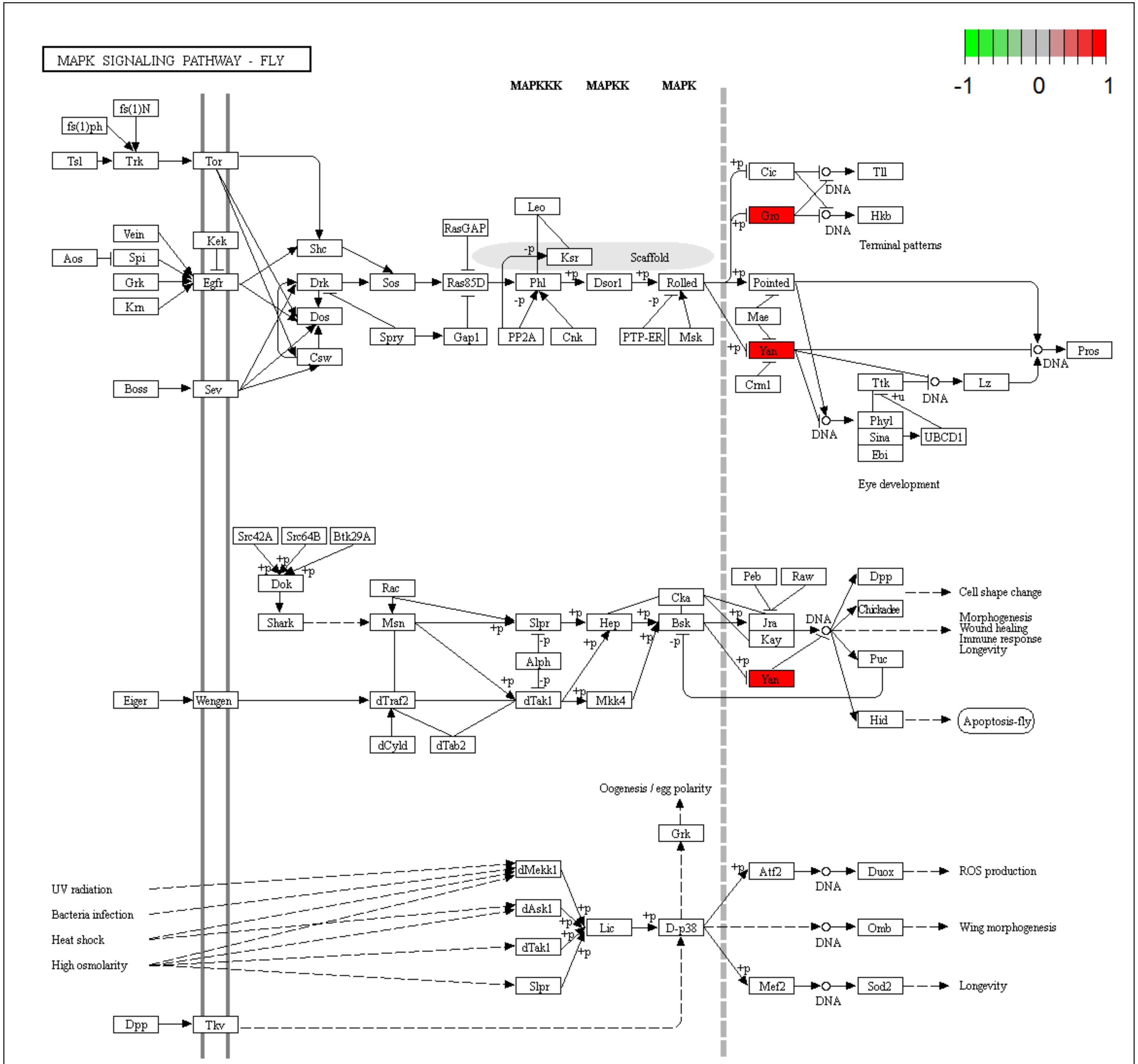

Data on KEGG graph
Rendered by Pathview

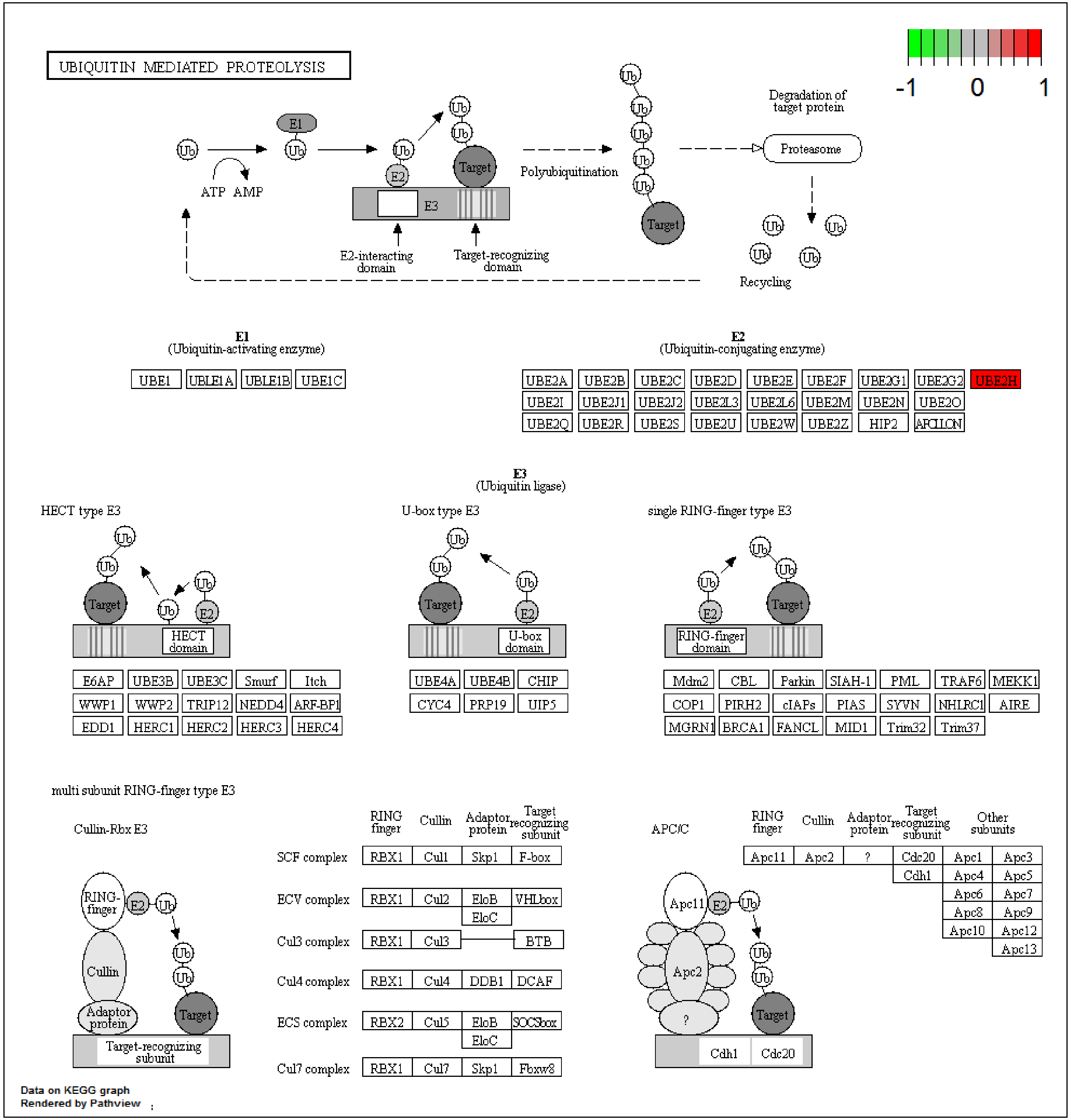

UBIQUITIN MEDIATED PROTEOLYSIS
-1 0 1
Ub
E1
ATP AMP
E2
E3
Target
E2-interacting domain
Target-recognizing domain
Polyubiquitination
Degradation of target protein
Proteasome
Recycling
E1
(Ubiquitin-activating enzyme)
UBE1 UBLE1A UBLE1B UBLE1C
E2
(Ubiquitin-conjugating enzyme)
UBE2A UBE2B UBE2C UBE2D UBE2E UBE2F UBE2G1 UBE2G2 UBE2H
UBE2I UBE2J1 UBE2J2 UBE2L3 UBE2L6 UBE2M UBE2N UBE2O
UBE2Q UBE2R UBE2S UBE2U UBE2W UBE2Z HIP2 APOLLON
E3
(Ubiquitin ligase)
HECT type E3
HECT domain
E6AP UBE3B UBE3C Smurf Itch
WWP1 WWP2 TRIP12 NEDD4 ARF-BP1
EDD1 HERC1 HERC2 HERC3 HERC4
U-box type E3
U-box domain
UBE4A UBE4B CHIP
CYC4 PRP19 UIP5
single RING-finger type E3
RING-finger domain
Mdm2 CBL Parkin SIAH-1 PML TRAF6 MEKK1
COP1 PIRH2 cIAPs PIAS SYVN NHLRC1 AIRE
MGRN1 BRCA1 FANCL MID1 Trim32 Trim37
multi subunit RING-finger type E3
Cullin-Rbx E3
RING-finger
Cullin
Adaptor protein
Target-recognizing subunit
RING finger Cullin Adaptor protein Target recognizing subunit
SCF complex RBX1 Cul1 Skp1 F-box
ECV complex RBX1 Cul2 EloB VHLbox EloC
Cul3 complex RBX1 Cul3 BTB
Cul4 complex RBX1 Cul4 DDB1 DCAF
ECS complex RBX2 Cul5 EloB SOCSbox EloC
Cul7 complex RBX1 Cul7 Skp1 Fbxw8
APC/C
RING finger Cullin Adaptor protein Target recognizing subunit Other subunits
Apc11 Apc2 ? Cdc20 Apc1 Apc3
Cdh1 Apc4 Apc5
Apc6 Apc7
Apc8 Apc9
Apc10 Apc12
Apc13
Apc11
Apc2
?
Cdh1 Cdc20
Data on KEGG graph
Rendered by Pathview

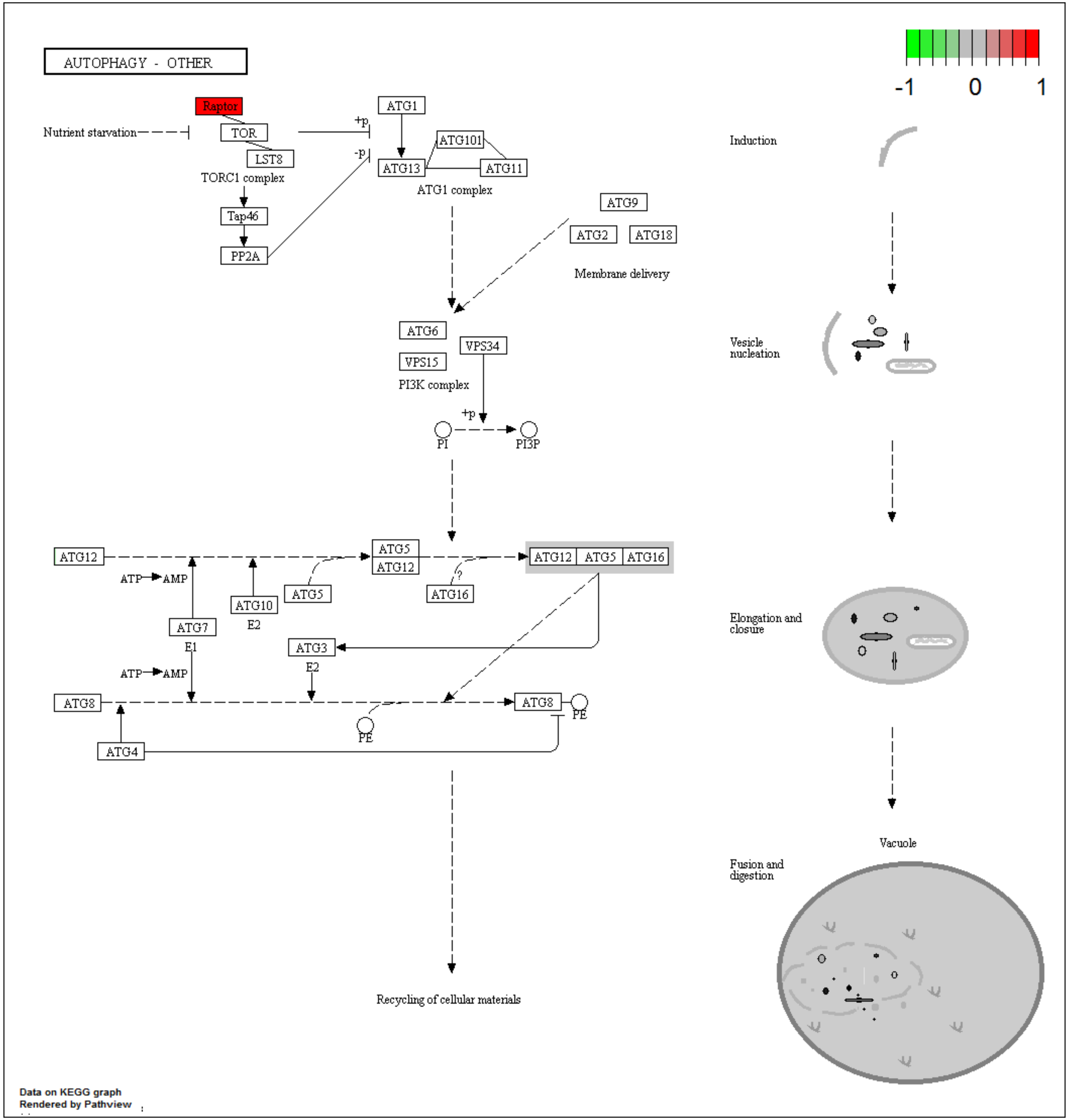
AUTOPHAGY - OTHER
-1
0
1
Raptor
TOR
LST8
Nutrient starvation
TORC1 complex
Tap46
PP2A
+p
-p
ATG1
ATG101
ATG13
ATG11
ATG1 complex
ATG9
ATG2
ATG18
Membrane delivery
ATG6
VPS34
VPS15
PI3K complex
+p
PI
PI3P
Induction
Vesicle nucleation
Elongation and closure
Vacuole
Fusion and digestion
ATG12
ATP
AMP
ATG5
ATG12
ATG12
ATG5
ATG16
ATG5
ATG16
ATG10
E2
ATG7
E1
ATG3
E2
ATG8
ATG8
PE
PE
ATG4
Recycling of cellular materials
Data on KEGG graph
Rendered by Pathview

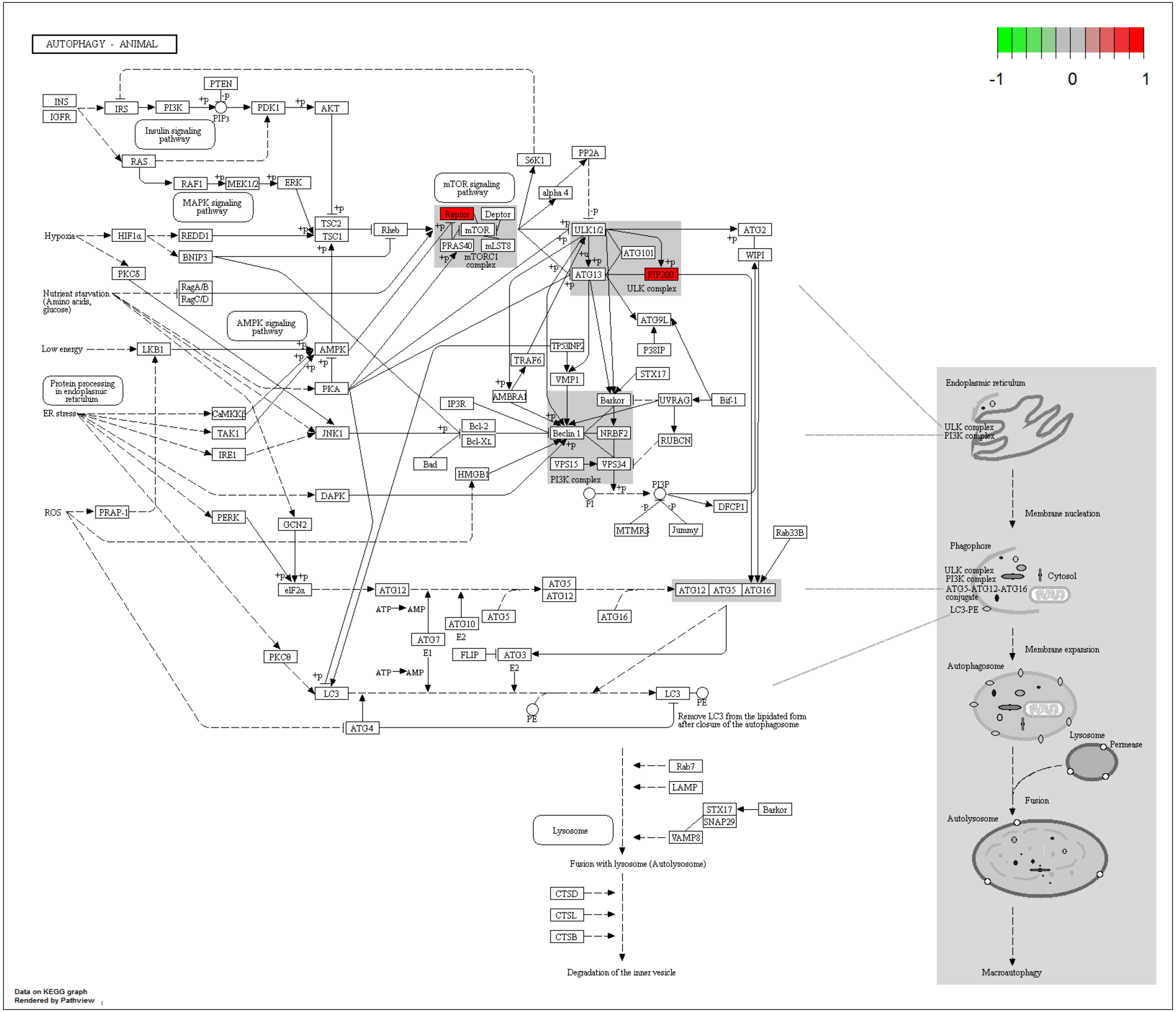
AUTOPHAGY - ANIMAL
-1
0
1
INS
IGFR
IRS
PI3K
PTEN
PIP3
PDK1
AKT
Insulin signaling pathway
RAS
RAF1
MEK1/2
ERK
MAPK signaling pathway
mTOR signaling pathway
Raptor
Deptor
mTOR
PRAS40
mLST8
mTORC1 complex
TSC2
TSC1
Rheb
Hypoxia
HIF1α
REDD1
BNIP3
PKCδ
Nutrient starvation (Amino acids, glucose)
RagA/B
RagC/D
AMPK signaling pathway
Low energy
LKB1
AMPK
PKA
Protein processing in endoplasmic reticulum
ER stress
CaMKKβ
TAK1
IRE1
JNK1
DAPK
PERK
GCN2
ROS
PRAP-1
eIF2α
S6K1
PP2A
alpha 4
ULK1/2
ATG101
ATG13
FIP200
ULK complex
ATG2
WIPI
ATG9L
P38IP
STX17
TP53INP2
TRAF6
VMP1
AMBRA1
IP3R
Bcl-2
Bcl-XL
Bad
HMGB1
Barkor
UVRAG
Bif-1
Beclin 1
NRBF2
RUBCN
VPS15
VPS34
PI3K complex
PI
PI3P
MTMR3
Jummy
DFCP1
Rab33B
ATG12
ATG5
ATG16
ATG10
ATG7
E1
E2
ATG3
FLIP
ATP
AMP
LC3
PE
ATG4
Remove LC3 from the lipidated form after closure of the autophagosome
Rab7
LAMP
STX17
SNAP29
VAMP8
Lysosome
Fusion with lysosome (Autolysosome)
CTSD
CTSL
CTSB
Degradation of the inner vesicle
Endoplasmic reticulum
ULK complex
PI3K complex
Membrane nucleation
Phagophore
ATG5-ATG12-ATG16 conjugate
LC3-PE
Cytosol
Membrane expansion
Autophagosome
Lysosome
Permease
Fusion
Autolysosome
Macroautophagy
Data on KEGG graph
Rendered by Pathview

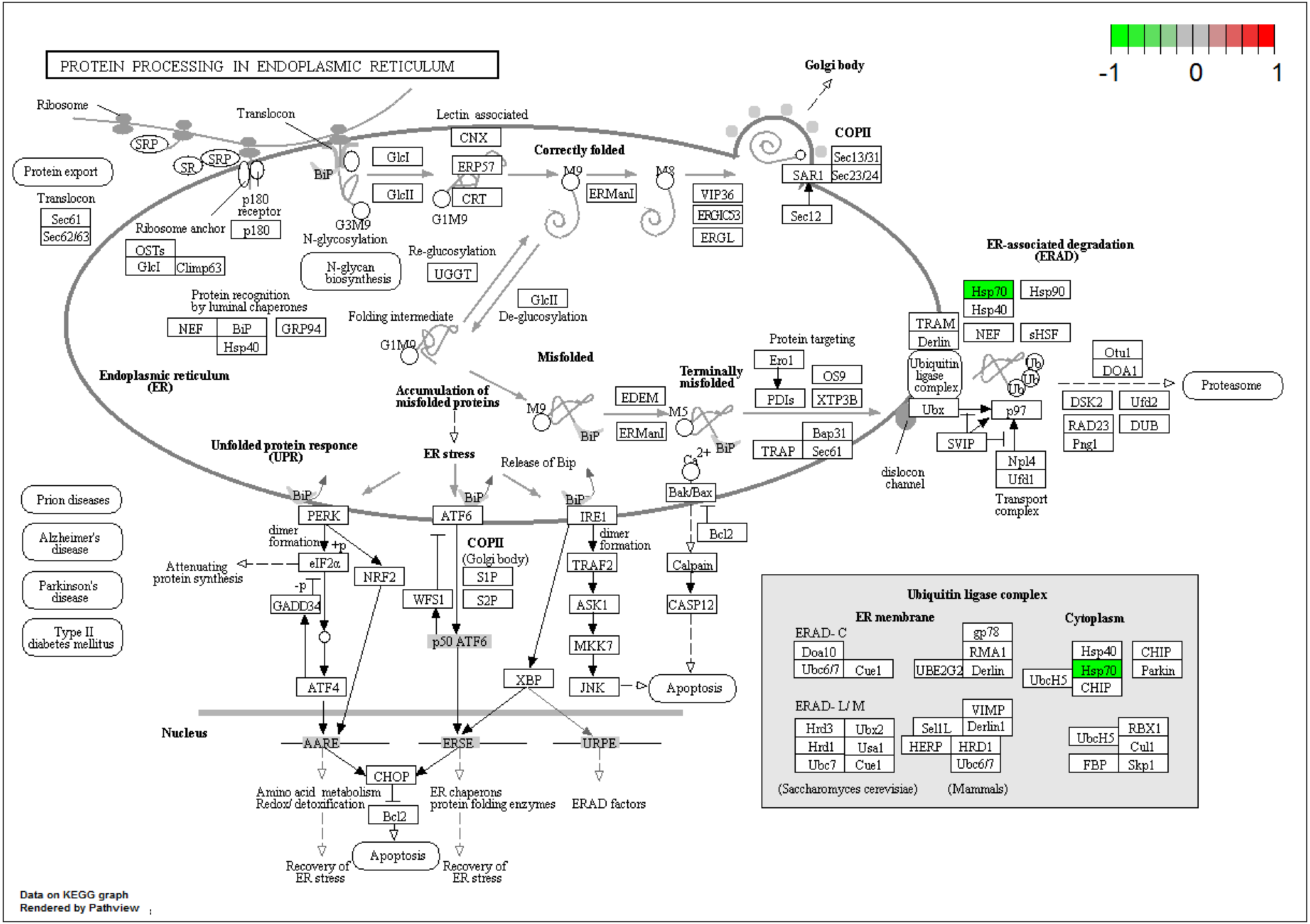
PROTEIN PROCESSING IN ENDOPLASMIC RETICULUM
-1
0
1
Golgi body
COPII
Sec13/31
Sec23/24
SAR1
Sec12
Ribosome
Translocon
SRP
SR
Protein export
p180 receptor
Sec61
Sec62/63
Ribosome anchor
p180
OSTs
GlcI
Climp63
BiP
G3M9
N-glycosylation
N-glycan biosynthesis
Lectin associated
CNX
GlcI
ERP57
GlcII
CRT
G1M9
Correctly folded
M9
M8
ERManI
VIP36
ERGIC53
ERGL
Re-glucosylation
UGGT
GlcII
De-glucosylation
Protein recognition by luminal chaperones
NEF
BiP
Hsp40
GRP94
Folding intermediate
Endoplasmic reticulum (ER)
Misfolded
Accumulation of misfolded proteins
EDEM
ERManI
M5
Terminally misfolded
Protein targeting
Ero1
OS9
PDIs
XTP3B
Bap31
TRAP
Sec61
ER-associated degradation (ERAD)
Hsp70
Hsp90
Hsp40
TRAM
Derlin
NEF
sHSF
Ubiquitin ligase complex
Ub
Otu1
DOA1
Proteasome
Ubx
p97
DSK2
Ufd2
RAD23
DUB
Png1
SVIP
Npl4
Ufd1
Transport complex
dislocon channel
Unfolded protein responce (UPR)
ER stress
Release of Bip
Ca2+
Bak/Bax
Bcl2
Calpain
CASP12
Prion diseases
Alzheimer's disease
Parkinson's disease
Type II diabetes mellitus
PERK
dimer formation
+p
eIF2α
-p
GADD34
Attenuating protein synthesis
NRF2
ATF4
ATF6
WFS1
COPII (Golgi body)
S1P
S2P
p50 ATF6
IRE1
dimer formation
TRAF2
ASK1
MKK7
JNK
Apoptosis
XBP
Nucleus
AARE
ERSE
URPE
CHOP
Amino acid metabolism
Redox/ detoxification
Bcl2
Apoptosis
ER chaperons
protein folding enzymes
ERAD factors
Recovery of ER stress
Recovery of ER stress
Ubiquitin ligase complex
ER membrane
Cytoplasm
ERAD- C
Doa10
Ubc6/7
Cue1
gp78
RMA1
UBE2G2
Derlin
Hsp40
Hsp70
CHIP
UbcH5
CHIP
Parkin
ERAD- L/ M
Hrd3
Ubx2
Hrd1
Usa1
Ubc7
Cue1
VIMP
Sel1L
Derlin1
HERP
HRD1
Ubc6/7
UbcH5
RBX1
Cul1
FBP
Skp1
(Saccharomyces cerevisiae)
(Mammals)
Data on KEGG graph
Rendered by Pathview

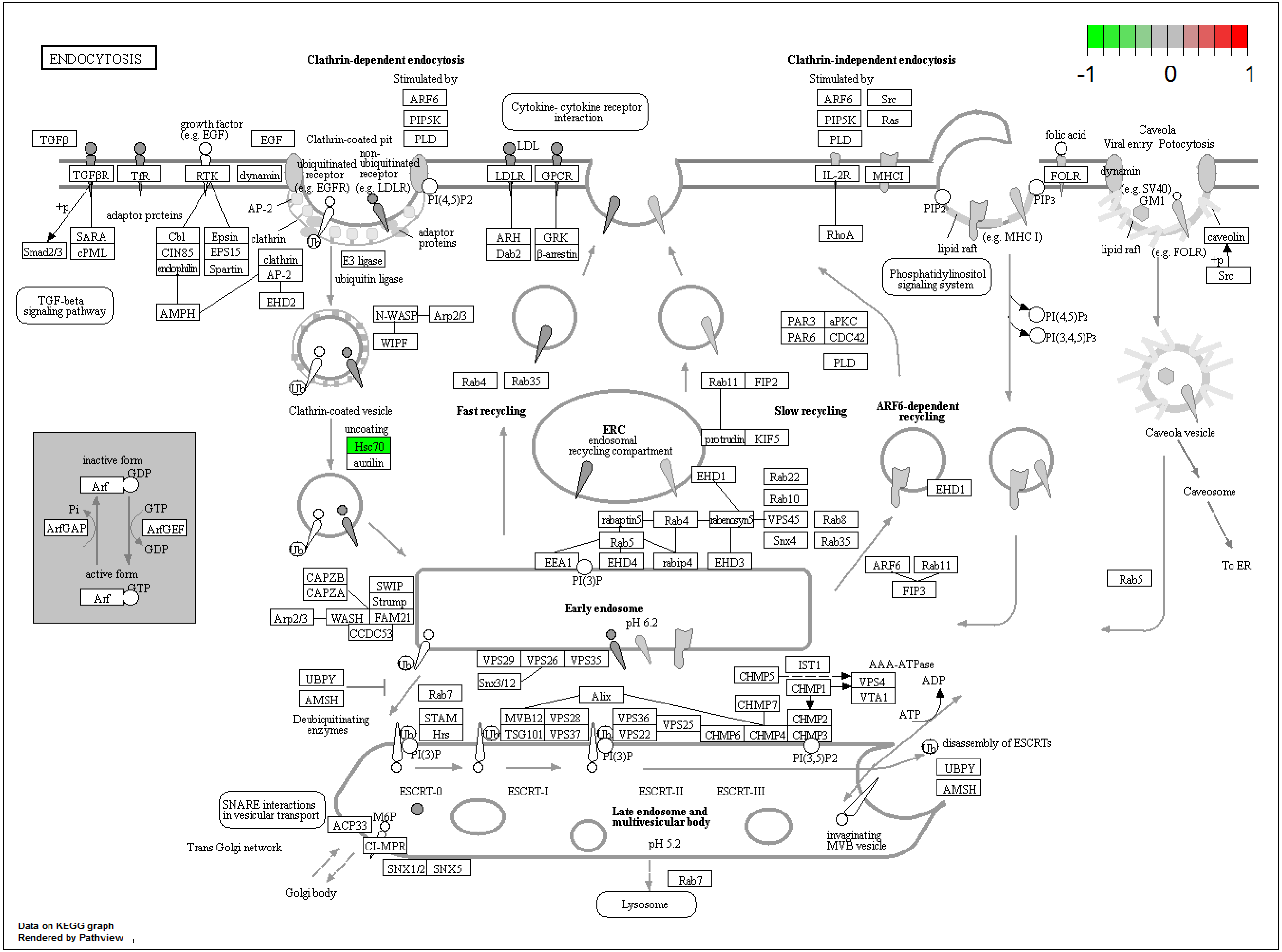
ENDOCYTOSIS
Clathrin-dependent endocytosis
Stimulated by
ARF6
PIP5K
PLD
Clathrin-independent endocytosis
Stimulated by
ARF6
Src
PIP5K
Ras
PLD
-1
0
1
Cytokine- cytokine receptor interaction
TGFβ
growth factor (e.g. EGF)
EGF
Clathrin-coated pit
LDL
folic acid
Caveola
Viral entry
Potocytosis
TGFβR
TfR
RTK
dynamin
non-ubiquitinated receptor (e.g. LDLR)
ubiquitinated receptor (e.g. EGFR)
LDLR
GPCR
IL-2R
MHCI
FOLR
dynamin
PI(4,5)P2
AP-2
+p
adaptor proteins
SARA
cPML
Smad2/3
Cbl
CIN85
endophilin
Epsin
EPS15
Spartin
clathrin
adaptor proteins
ARH
Dab2
GRK
β-arrestin
RhoA
PIP2
PIP3
(e.g. SV40)
GM1
lipid raft
(e.g. MHC I)
lipid raft
(e.g. FOLR)
caveolin
+p
Src
clathrin
AP-2
EHD2
E3 ligase
ubiquitin ligase
TGF-beta signaling pathway
AMPH
N-WASP
Arp2/3
WIPF
Phosphatidylinositol signaling system
PAR3
aPKC
PAR6
CDC42
PLD
PI(4,5)P2
PI(3,4,5)P3
Rab4
Rab35
Rab11
FIP2
Clathrin-coated vesicle
Fast recycling
Slow recycling
ARF6-dependent recycling
Caveola vesicle
uncoating
Hsc70
auxilin
ERC
endosomal recycling compartment
protrudin
KIF5
EHD1
Rab22
Rab10
EHD1
Caveosome
inactive form
GDP
Arf
Pi
GTP
ArfGAP
ArfGEF
GDP
active form
GTP
Arf
rabaptin5
Rab4
rabenosyn5
VPS45
Rab8
Rab5
Snx4
Rab35
EEA1
EHD4
rabip4
EHD3
ARF6
Rab11
FIP3
To ER
Rab5
PI(3)P
CAPZB
CAPZA
SWIP
Strump
Arp2/3
WASH
FAM21
CCDC53
Early endosome
pH 6.2
VPS29
VPS26
VPS35
Snx3/12
IST1
CHMP5
AAA-ATPase
CHMP1
VPS4
VTA1
ADP
UBPY
AMSH
Rab7
Alix
CHMP7
ATP
Deubiquitinating enzymes
STAM
Hrs
MVB12
VPS28
TSG101
VPS37
VPS36
VPS22
VPS25
CHMP6
CHMP4
CHMP2
CHMP3
disassembly of ESCRTs
PI(3)P
PI(3)P
PI(3,5)P2
UBPY
AMSH
ESCRT-0
ESCRT-I
ESCRT-II
ESCRT-III
SNARE interactions in vesicular transport
ACP33
M6P
Late endosome and multivesicular body
invaginating MVB vesicle
CI-MPR
pH 5.2
Trans Golgi network
SNX1/2
SNX5
Rab7
Golgi body
Lysosome
Data on KEGG graph
Rendered by Pathview

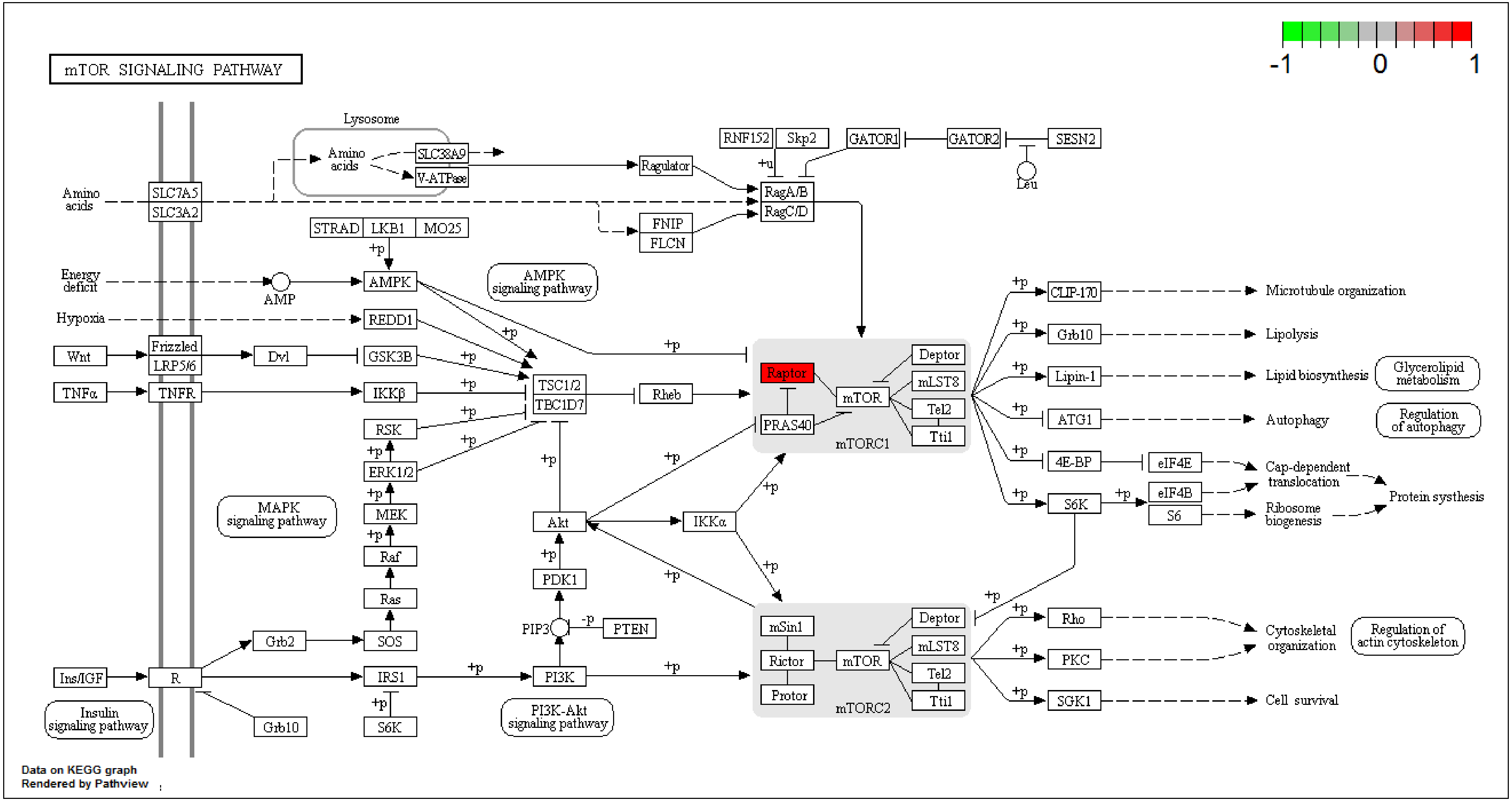

mTOR SIGNALING PATHWAY
-1
0
1
Lysosome
Amino acids
SLC38A9
V-ATPase
Ragulator
RNF152
Skp2
GATOR1
GATOR2
SESN2
Leu
RagA/B
RagC/D
SLC7A5
SLC3A2
FNIP
FLCN
STRAD
LKB1
MO25
Energy deficit
AMP
AMPK
AMPK signaling pathway
Hypoxia
REDD1
Wnt
Frizzled
LRP5/6
Dvl
GSK3B
TNFα
TNFR
IKKβ
RSK
ERK1/2
MEK
Raf
Ras
SOS
Grb2
TSC1/2
TBC1D7
Rheb
Raptor
PRAS40
mTOR
Deptor
mLST8
Tel2
Tti1
mTORC1
MAPK signaling pathway
Akt
IKKα
PDK1
PIP3
PTEN
PI3K
IRS1
S6K
Grb10
Ins/IGF
R
Insulin signaling pathway
PI3K-Akt signaling pathway
mSin1
Rictor
Protor
mTORC2
CLIP-170
Microtubule organization
Lipolysis
Lipin-1
Lipid biosynthesis
Glycerolipid metabolism
ATG1
Autophagy
Regulation of autophagy
4E-BP
eIF4E
Cap-dependent translocation
eIF4B
S6
Ribosome biogenesis
Protein systhesis
Rho
PKC
SGK1
Cytoskeletal organization
Regulation of actin cytoskeleton
Cell survival
Data on KEGG graph
Rendered by Pathview

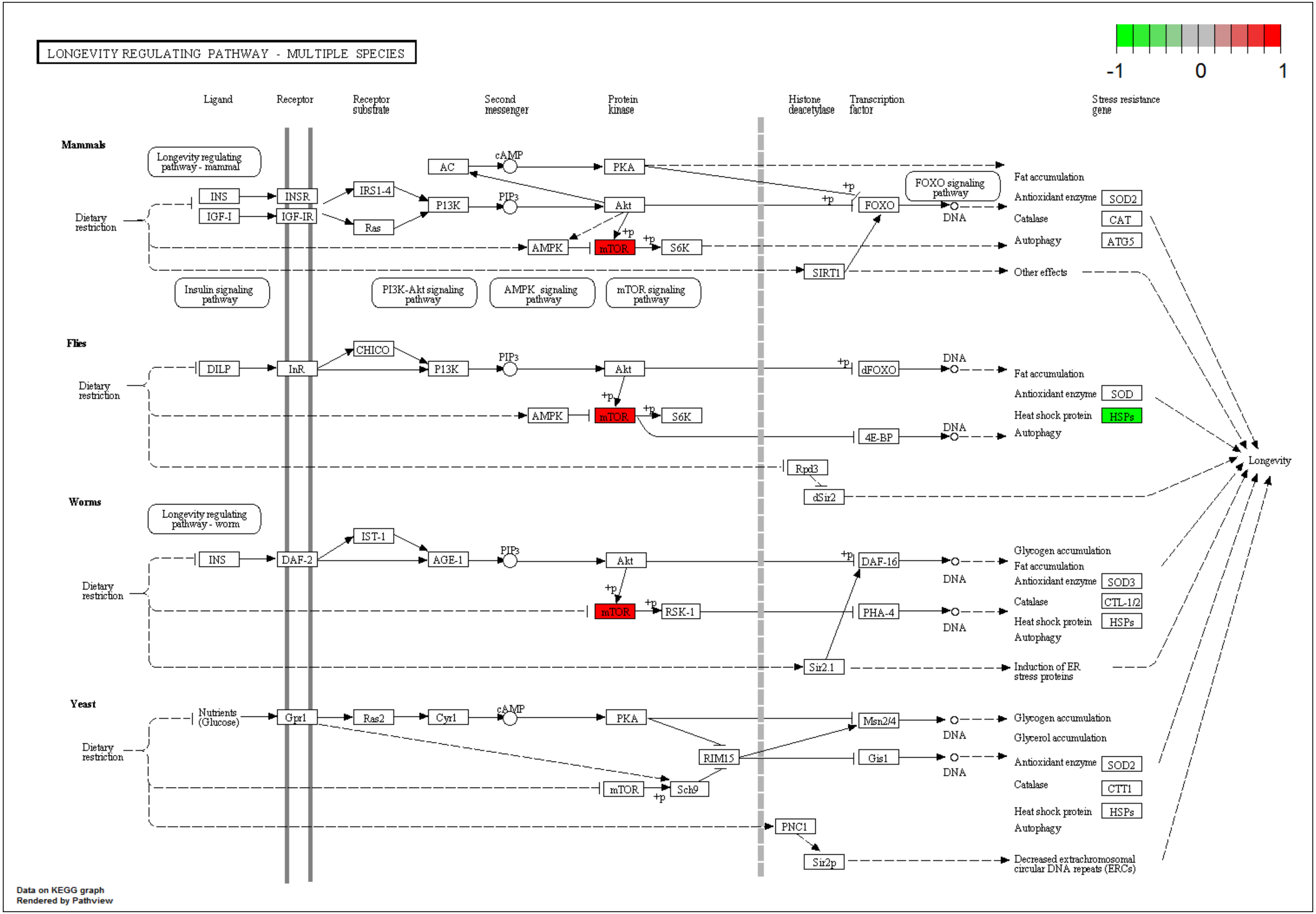

LONGEVITY REGULATING PATHWAY - MULTIPLE SPECIES
-1
0
1
Ligand
Receptor
Receptor substrate
Second messenger
Protein kinase
Histone deacetylase
Transcription factor
Stress resistance gene
Mammals
Longevity regulating pathway - mammal
Dietary restriction
INS
IGF-I
INSR
IGF-IR
IRS1-4
Ras
AC
cAMP
PKA
P13K
PIP3
Akt
AMPK
mTOR
S6K
SIRT1
FOXO
FOXO signaling pathway
DNA
+p
Fat accumulation
Antioxidant enzyme
SOD2
Catalase
CAT
Autophagy
ATG5
Other effects
Insulin signaling pathway
PI3K-Akt signaling pathway
AMPK signaling pathway
mTOR signaling pathway
Flies
Dietary restriction
DILP
InR
CHICO
P13K
PIP3
Akt
AMPK
mTOR
S6K
dFOXO
4E-BP
Rpd3
dSir2
DNA
Fat accumulation
Antioxidant enzyme
SOD
Heat shock protein
HSPs
Autophagy
Longevity
Worms
Longevity regulating pathway - worm
Dietary restriction
INS
DAF-2
IST-1
AGE-1
PIP3
Akt
mTOR
RSK-1
DAF-16
PHA-4
Sir2.1
DNA
Glycogen accumulation
Fat accumulation
Antioxidant enzyme
SOD3
Catalase
CTL-1/2
Heat shock protein
HSPs
Autophagy
Induction of ER stress proteins
Yeast
Dietary restriction
Nutrients (Glucose)
Gpr1
Ras2
Cyr1
cAMP
PKA
RIM15
mTOR
Sch9
Msn2/4
Gis1
PNC1
Sir2p
DNA
Glycogen accumulation
Glycerol accumulation
Antioxidant enzyme
SOD2
Catalase
CTT1
Heat shock protein
HSPs
Autophagy
Decreased extrachromosomal circular DNA repeats (ERCs)
Data on KEGG graph
Rendered by Pathview

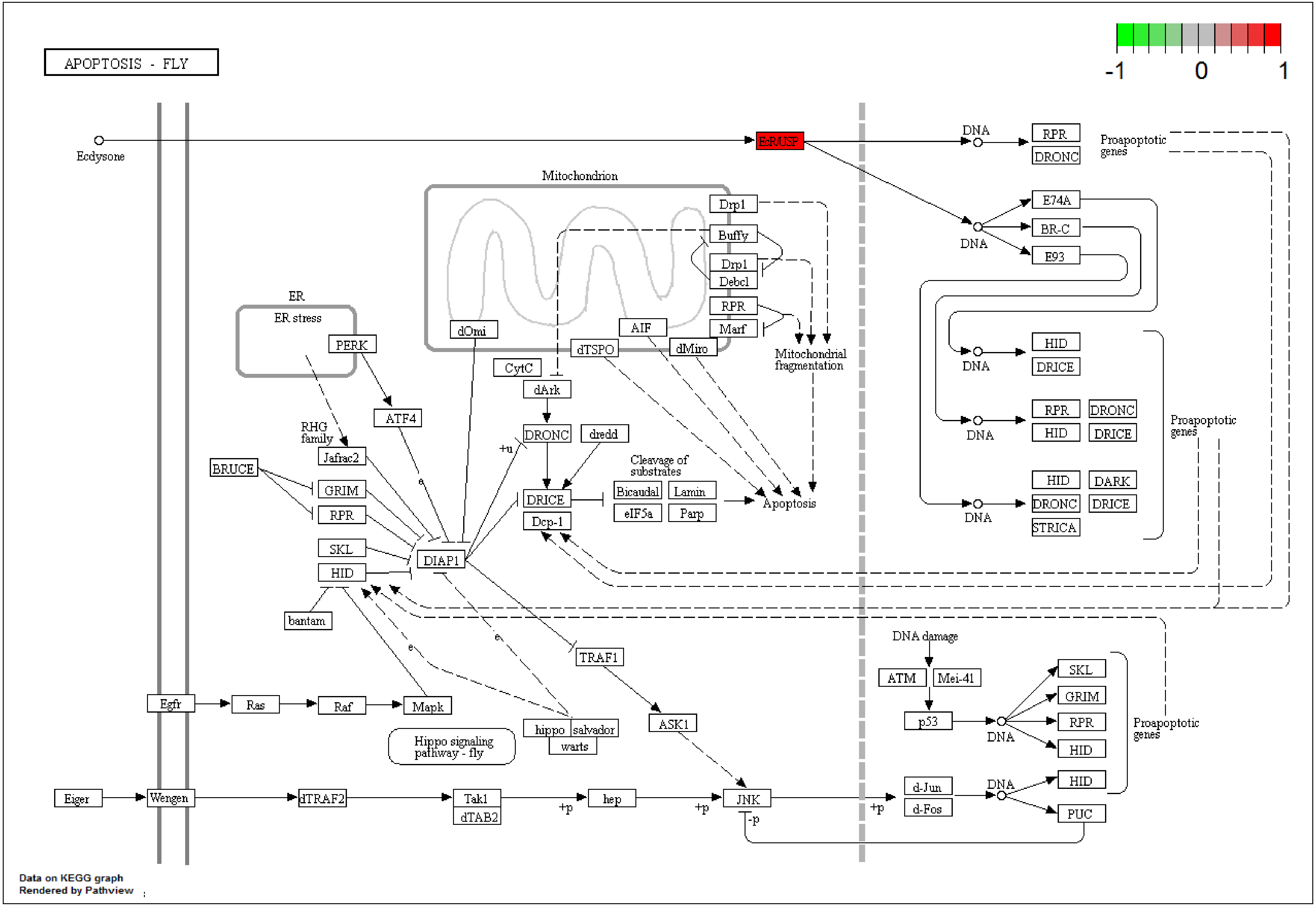
APOPTOSIS - FLY
-1
0
1
Ecdysone
EcR/USP
DNA
RPR
DRONC
Proapoptotic genes
Mitochondrion
Drp1
Buffy
Drp1
Debcl
RPR
Marf
E74A
BR-C
E93
DNA
ER
ER stress
PERK
dOmi
AIF
dTSPO
dMiro
CytC
Mitochondrial fragmentation
HID
DRICE
DNA
dArk
ATF4
RHG family
DRONC
dredd
RPR
DRONC
HID
DRICE
DNA
Proapoptotic genes
Jafrac2
BRUCE
+u
Cleavage of substrates
GRIM
DRICE
Bicaudal
Lamin
Apoptosis
HID
DARK
RPR
eIF5a
Parp
DRONC
DRICE
DNA
Dcp-1
STRICA
SKL
DIAP1
HID
bantam
DNA damage
TRAF1
ATM
Mei-41
SKL
GRIM
Egfr
Ras
Raf
Mapk
hippo
salvador
ASK1
p53
RPR
DNA
warts
Proapoptotic genes
Hippo signaling pathway - fly
HID
HID
Eiger
Wengen
dTRAF2
Tak1
dTAB2
hep
JNK
+p
+p
+p
-p
d-Jun
d-Fos
DNA
PUC
Data on KEGG graph
Rendered by Pathview

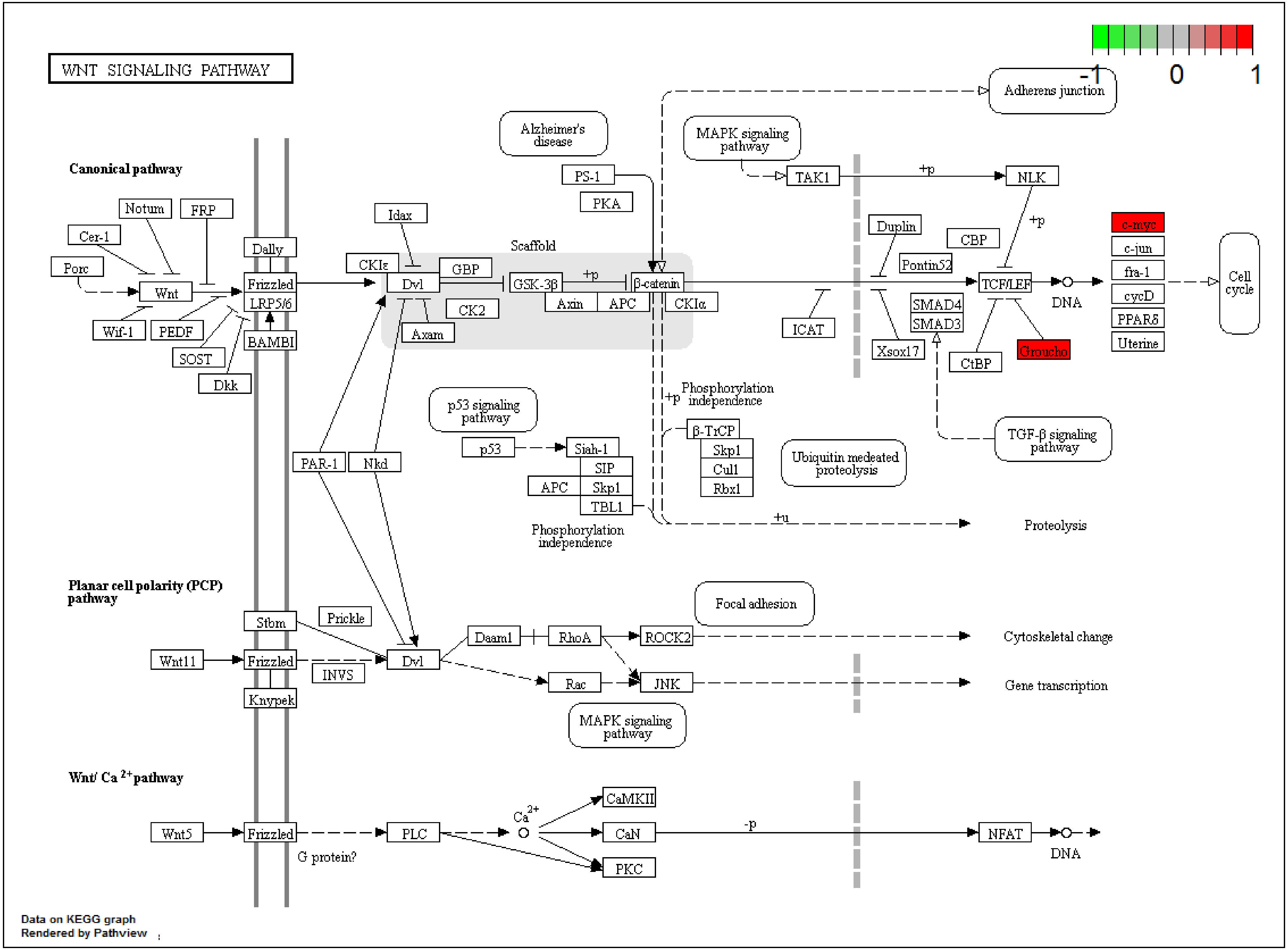

WNT SIGNALING PATHWAY
-1
0
1
Canonical pathway
Notum
FRP
Cer-1
Porc
Wnt
Wif-1
PEDF
SOST
Dkk
Dally
Frizzled
LRP5/6
BAMBI
Idax
CKIε
Dvl
GBP
CK2
Axam
Scaffold
Alzheimer's disease
PS-1
PKA
GSK-3β
+p
Axin
APC
β-catenin
CKIα
MAPK signaling pathway
TAK1
+p
NLK
Adherens junction
Duplin
CBP
Pontin52
+p
ICAT
SMAD4
SMAD3
Xsox17
TCF/LEF
CtBP
Groucho
DNA
c-myc
c-jun
fra-1
cycD
PPARδ
Uterine
Cell cycle
+p
Phosphorylation independence
β-TrCP
Skp1
Cul1
Rbx1
Ubiquitin mediated proteolysis
TGF-β signaling pathway
p53 signaling pathway
p53
Siah-1
SIP
APC
Skp1
TBL1
Phosphorylation independence
+u
Proteolysis
PAR-1
Nkd
Planar cell polarity (PCP) pathway
Stbm
Prickle
Wnt11
Frizzled
INVS
Knypek
Dvl
Daam1
RhoA
ROCK2
Focal adhesion
Cytoskeletal change
Rac
JNK
Gene transcription
MAPK signaling pathway
Wnt/ Ca 2+ pathway
Wnt5
Frizzled
PLC
G protein?
Ca2+
CaMKII
CaN
PKC
-p
NFAT
DNA
Data on KEGG graph
Rendered by Pathview

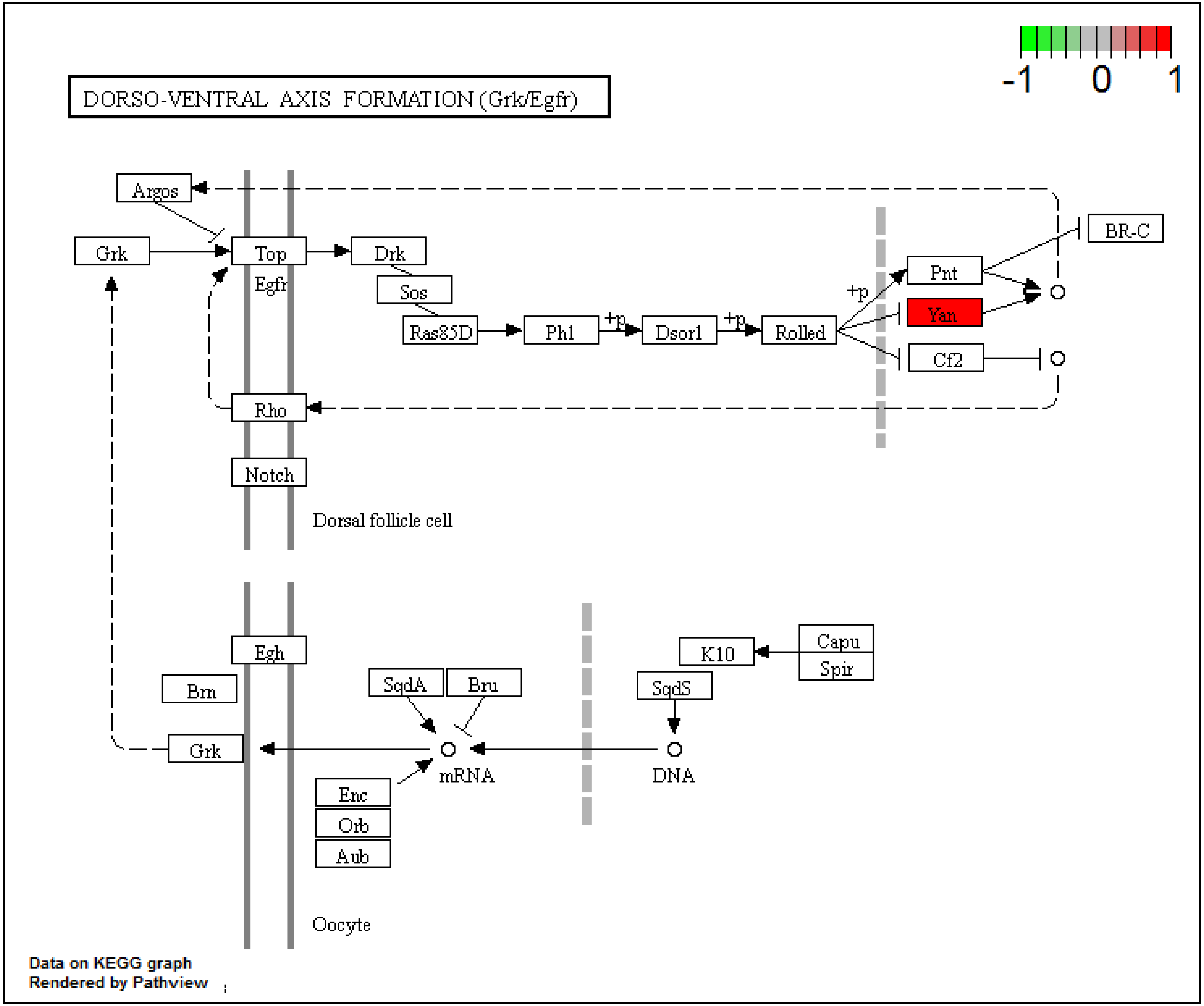

-1
0
1
DORSO-VENTRAL AXIS FORMATION (Grk/Egfr)
Argos
Grk
Top
Egfr
Drk
Sos
Ras85D
Phl
+p
Dsor1
+p
Rolled
+p
Pnt
Yan
Cf2
BR-C
Rho
Notch
Dorsal follicle cell
Egh
Brn
Grk
SqdA
Bru
mRNA
Enc
Orb
Aub
K10
Capu
Spir
SqdS
DNA
Oocyte
Data on KEGG graph
Rendered by Pathview

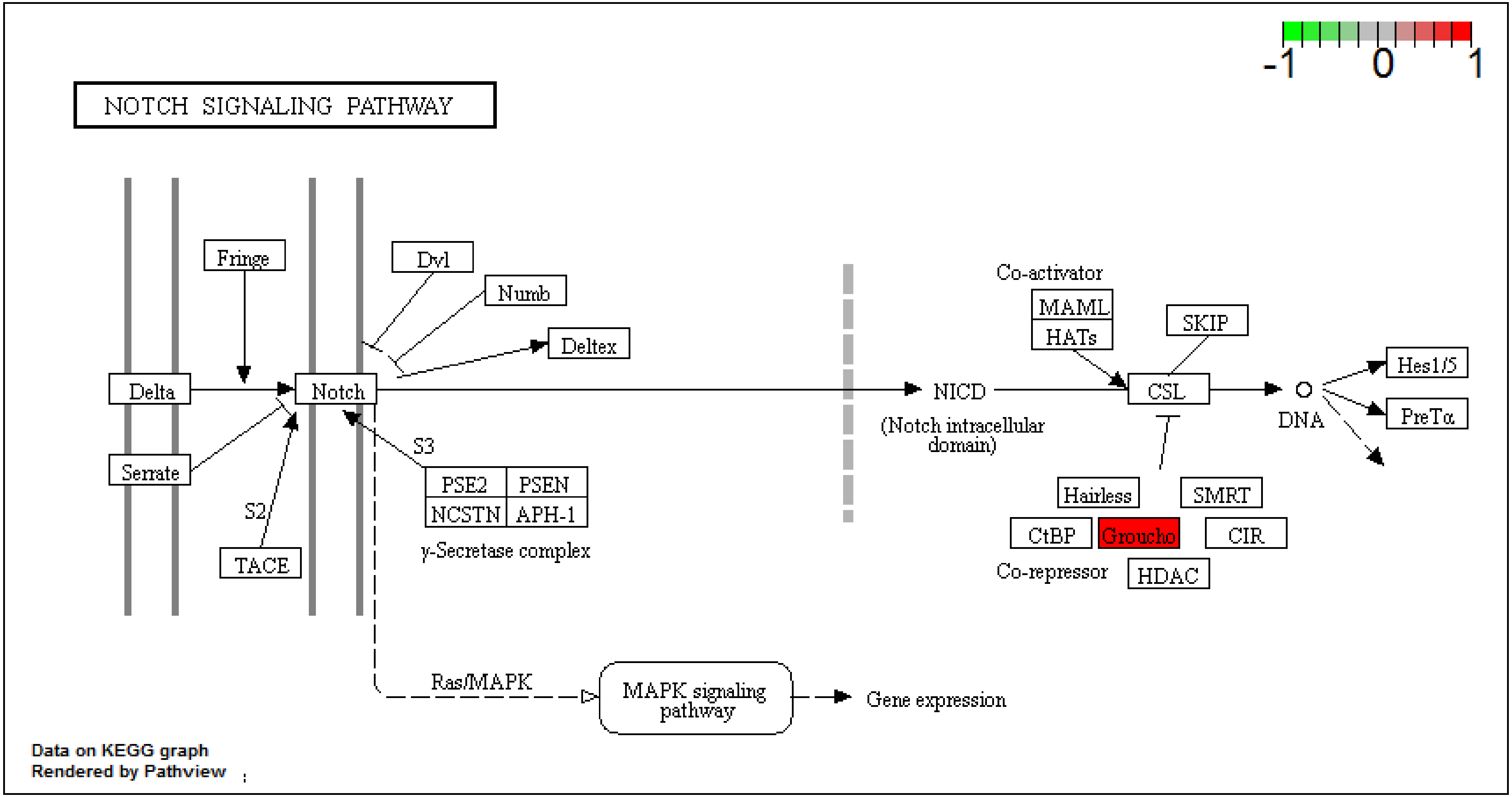

-1
0
1
NOTCH SIGNALING PATHWAY
Fringe
Dvl
Numb
Deltex
Delta
Notch
NICD
(Notch intracellular domain)
Co-activator
MAML
HATs
SKIP
CSL
DNA
Hes1/5
PreTα
Serrate
S2
TACE
S3
PSE2
PSEN
NCSTN
APH-1
γ-Secretase complex
Hairless
SMRT
CtBP
Groucho
CIR
Co-repressor
HDAC
Ras/MAPK
MAPK signaling pathway
Gene expression
Data on KEGG graph
Rendered by Pathview

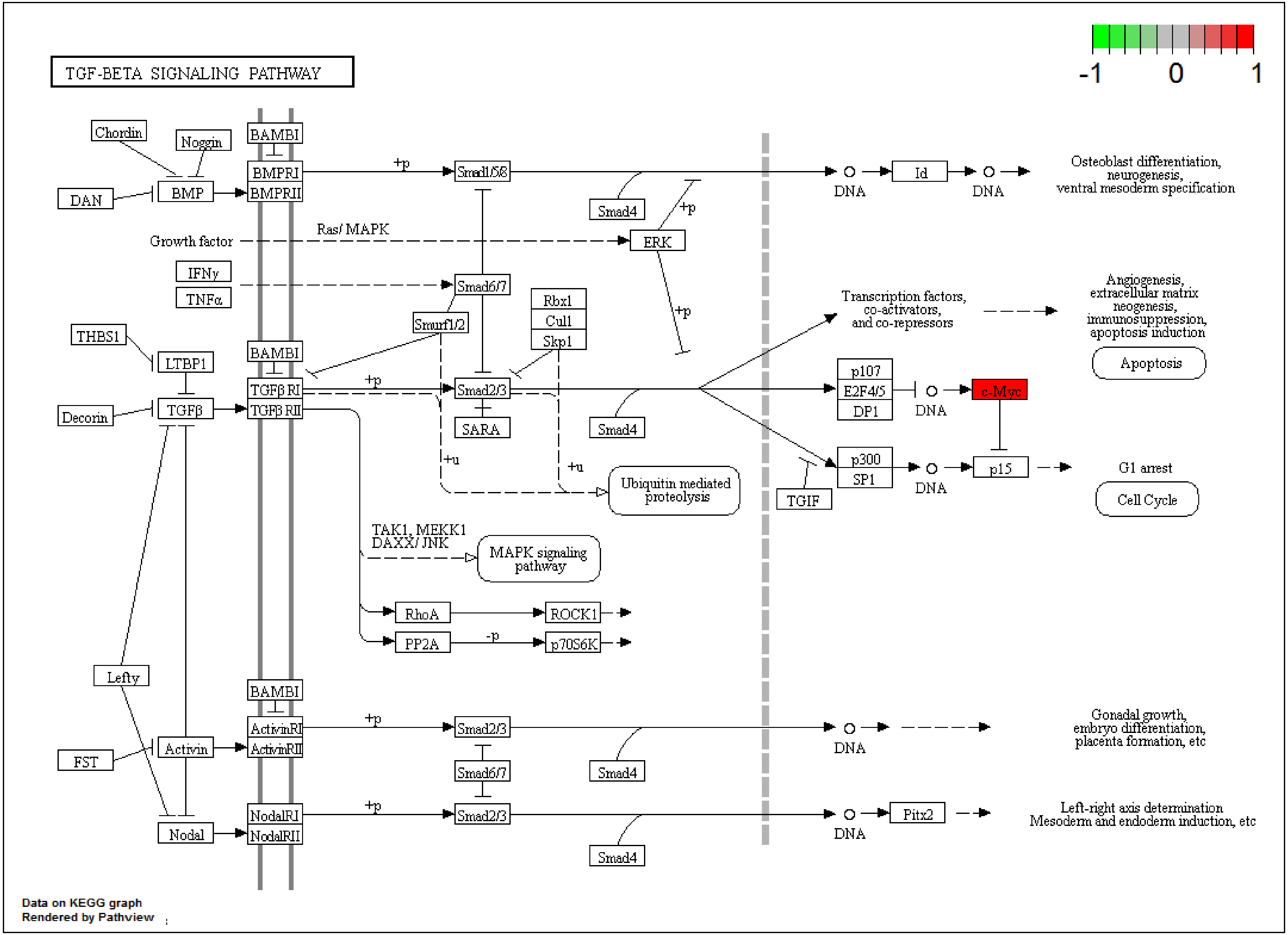
TGF-BETA SIGNALING PATHWAY
-1
0
1
Chordin
Noggin
DAN
BMP
BAMBI
BMPRI
BMPRII
+p
Smad1/5/8
Smad4
+p
DNA
Id
DNA
Osteoblast differentiation, neurogenesis, ventral mesoderm specification
Growth factor
Ras/ MAPK
ERK
IFNγ
TNFα
Smad6/7
+p
Smurf1/2
Rbx1
Cul1
Skp1
THBS1
LTBP1
BAMBI
Transcription factors, co-activators, and co-repressors
Angiogenesis, extracellular matrix neogenesis, immunosuppression, apoptosis induction
Apoptosis
TGFβ RI
TGFβ RII
TGFβ
Decorin
+p
Smad2/3
SARA
Smad4
p107
E2F4/5
DP1
DNA
c-Myc
p300
SP1
DNA
p15
G1 arrest
Cell Cycle
TGIF
+u
+u
Ubiquitin mediated proteolysis
TAK1, MEKK1
DAXX/ JNK
MAPK signaling pathway
RhoA
ROCK1
PP2A
-p
p70S6K
Lefty
BAMBI
ActivinRI
ActivinRII
Activin
FST
+p
Smad2/3
Smad6/7
Smad4
DNA
Gonadal growth, embryo differentiation, placenta formation, etc
NodalRI
NodalRII
Nodal
+p
Smad2/3
Smad4
DNA
Pitx2
Left-right axis determination
Mesoderm and endoderm induction, etc
Data on KEGG graph
Rendered by Pathview

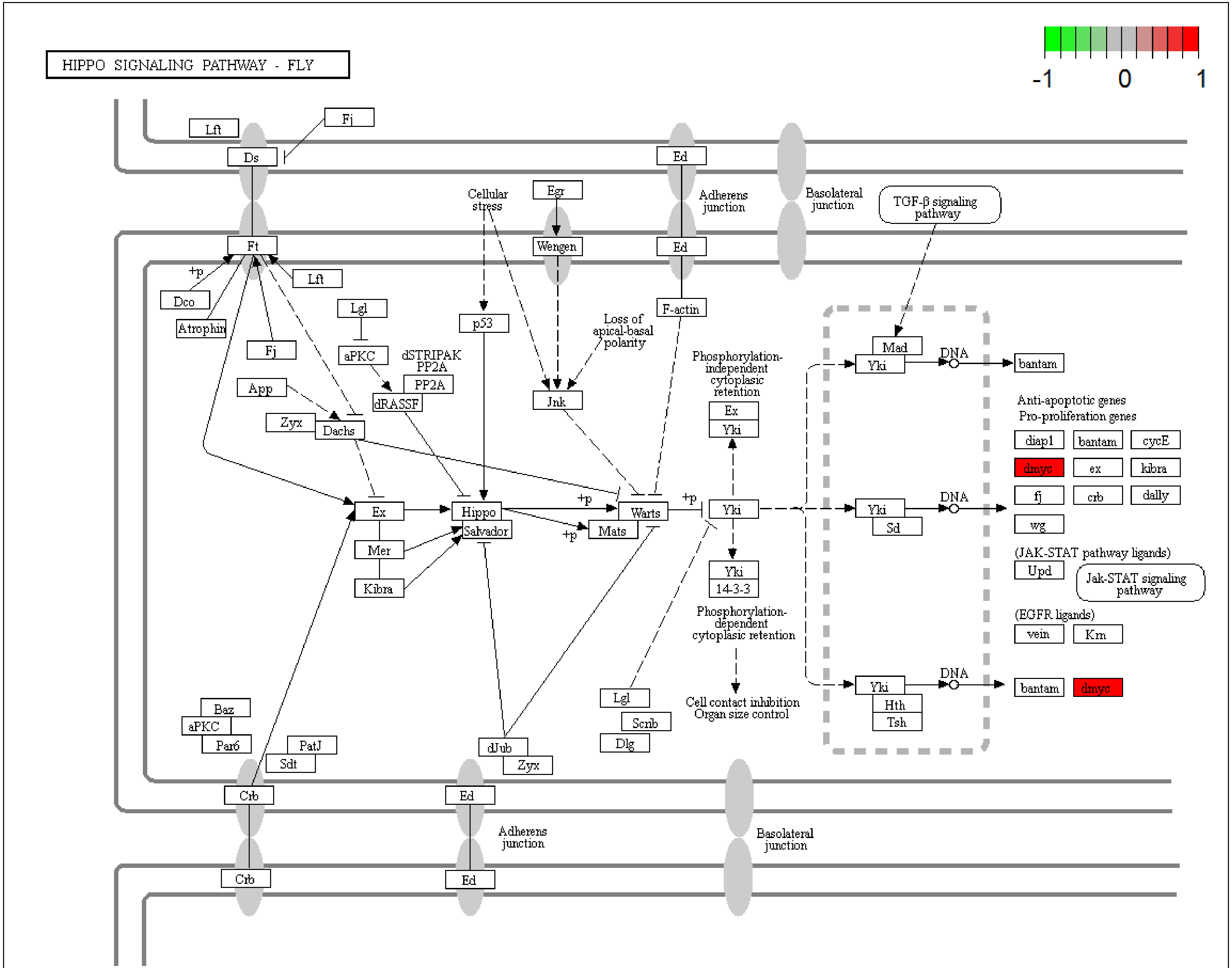

Data on KEGG graph
Rendered by Pathview
